# Complete Genome Characterization of Reticuloendotheliosis Virus Detected in Chickens with Multiple Viral Coinfections

**DOI:** 10.3390/v14040798

**Published:** 2022-04-13

**Authors:** Ruy D. Chacón, Benjy Sedano-Herrera, Elizabeth Regina Alfaro-Espinoza, Wilma Ursula Quispe, Arturo Liñan-Torres, David De la Torre, Anderson de Oliveira, Claudete S. Astolfi-Ferreira, Antonio J. Piantino Ferreira

**Affiliations:** 1 Department of Pathology, School of Veterinary Medicine, University of São Paulo, Av. Prof. Orlando M. Paiva, 87, São Paulo 05508-270, Brazil; ruychaconv@usp.br (R.D.C.); daviddelatorreduque@gmail.com (D.D.l.T.); aoliveira@usp.br (A.d.O.); csastolfi@gmail.com (C.S.A.-F.); 2 Inter-Units Program in Biotechnology, University of São Paulo, Av. Prof. Dr. Orlando M. Paiva, 87, São Paulo 05508-270, Brazil; 3 Departamento de Ecología, Museo de Historia Natural de la Universidad Nacional Mayor de San Marcos, Lima 15072, Peru; benjy.sedano@unmsm.edu.pe; 4 Asociación Peruana de Astrobiología-ASPAST, Lima, Peru; 5 Programa de Pós-Graduação em Bioinformática, Instituto de Ciências Biológicas, Universidade Federal de Minas Gerais, Belo Horizonte 31270-901, Brazil; elizaespinoza@ufmg.br; 6 Laboratory of Molecular Microbiology and Biotechnology, Faculty of Biological sciences, Universidad Nacional Mayor de San Marcos, Lima 15081, Peru; wilma.quispe@unmsm.edu.pe; 7 Instituto de Biotecnología, Universidad Nacional Autónoma de México, Av. Universidad 2001, Col. Chamilpa, Cuernavaca 62210, Morelos, Mexico; arturo.linan@ibt.unam.mx; 8 LABIGEN, Laboratory of Molecular Biology and Genetics, Quito EC170521, Ecuador

**Keywords:** reticuloendotheliosis virus (REV), viral coinfection, complete genome sequencing, phylogenetic analysis, protein modeling

## Abstract

Reticuloendotheliosis virus (REV) is a retroviral pathogen capable of infecting several avian hosts and is associated with immunosuppression, anemia, proventriculitis, neoplasia, and runting–stunting syndrome. Its genome contains the three major genes, *gag*, *pol*, and *env*, and two flanking long terminal repeat (LTR) regions. Complete genome sequences of REV are limited in terms of geographical origin. The aim of this study was to characterize the complete genome of REV detected in Brazilian chickens with multiple viral coinfections and analyze the polymorphisms in the deduced amino acids sequences corresponding to its encoded proteins. We tested the presence and completeness of REV as well as other viral pathogens in samples from Brazilian poultry farms by qPCR. The complete genomes of two REV strains were sequenced by overlapping fragments through the dideoxy method. Phylogenetic analysis, pairwise identity matrix, polymorphism identification and protein modeling were performed along the entire genome. We detected REV in 65% (26/40) of the tested samples. Concomitant viral infections were detected in 82.5% (33/40) of the samples and in 90% (9/10) of the farms. Multiple infections included up to seven viruses. Phylogenetic analysis classified both Brazilian strains into REV subtype 3, and the pairwise comparison indicated that strains from the USA and fowlpox virus (FWPV)-related strains were the most identical. The subdomain p18 in *gag*, the reverse transcriptase/ribonuclease H in *pol*, and the surface (SU) in the *env* protein were the most polymorphic in genomic comparisons. The relevant motifs for each protein were highly conserved, with fewer polymorphisms in the fusion peptide, immunosuppression domain, and disulfide bonds on the surface (SU) and transmembrane (TM) of *env*. This is the first study to include complete genomes of REV in Brazil and South America detected in farms with multiple viral coinfections. Our findings suggest an involvement of REV as an immunosuppressor and active agent in the emergence and progression of multiple infectious diseases. We also found a possible etiological relationship between Brazilian strains and the USA and FWPV recombinant strains. This information highlights the need for epidemiological vigilance regarding REV in association with another pathogens.

## 1. Introduction

Reticuloendotheliosis virus (REV) belongs to the *Retroviridae* family, the subfamily *Orthoretrovirinae* and the genus *Gammaretrovirus*. The spherical and enveloped REV virions have a diameter of 100 nm and display surface projections of 6 nm long, and 10 nm in diameter [1]. The REV genome is positive-sense single-stranded RNA and is near 8.3 kb in size. It includes three open reading frames (ORFs) encoding the group-specific antigen (*gag*), polymerase (*pol*), and envelope (*env*) genes. The *gag* gene comprises structural proteins of the virus: a matrix (MA), p18, capsid protein (CA), and nucleocapsid (NC), which are involved in viral particle assembly and encapsidation. *Pol* encodes the viral enzymes protease (PR), integrase (IN), and reverse transcriptase (RT), which are essential for viral replication. *Env* encodes the surface (SU) and transmembrane (TM) glycoproteins, whose critical function is to mediate the adsorption and the penetration of host cells susceptible to infection [1,2]. REV uses a neutral amino acid transporter (SLC1A5) to bind the host cell and is mediated by SU [2]. The long terminal repeats (LTRs) are identical non-coding sequences and flank the REV provirus.

REV infects several kinds of birds and has been studied because of its harmful impact on the global poultry industry. The prototype REV strain (REV-T) was isolated from a turkey in 1957 [3]. REV-T is a defective strain and carries an oncogene that is responsible for its acute oncogenicity [4]. Since then, other non-defective strains have been reported in birds such as ducks, chickens, geese, turkeys, peafowls, prairie chickens, pigeons, pheasants, and Japanese quails [5]. These non-defective strains are associated with a range of disease manifestations, including anemia, proventriculitis, immunosuppression, neoplasia, and runting–stunting syndrome [1].

REV tropism was observed for kidneys, lymphoid tissues, pancreas, blood cells and proventriculus, especially on epithelial cells [6]. REV can be transmitted horizontally by close contact with infected birds, contaminated environment, and insects, or vertically in a low frequency [5]. In commercial poultry is usually controlled by strict biosecurity and elimination of contaminated breeders, although some vaccines can generate some protection against infection [1]. REV belongs to a single serotype, but three antigenic subtypes have been identified [7].

Replication of REV genome after viral entry requires an initial RNA reverse transcription followed by an integration of the proviral DNA into the host cell genome [1]. REV provirus genomes have been identified as embedded within some DNA viruses of birds, such as a fowlpox virus (FWPV) [8], and Marek’s disease virus (MDV) [9]. Reports of viral coinfections including REV are scarce, mainly limited checking for oncogenic viruses with evidence of synergistic pathogenic effect [10,11]. In experimental studies of coinfections with bacteria or virus, the immunosuppression role was attributed to REV, even reducing the efficacy of vaccines [12,13].

Brazil is one of the largest producers and exporters in the poultry industry. Previous studies have reported the presence of REV in Brazil in coinfection with MDV, FWPV, or solely [14,15,16]. Nonetheless, no complete REV genome sequences have been reported neither in Brazil nor in South America. Moreover, there are a lack of reports of REV in coinfections with different viral pathogens.

The aim of this study was to characterize the complete genome of REV detected in Brazilian chickens with multiple viral coinfections and analyze the polymorphisms in the deduced amino acids sequences corresponding to its encoded proteins. This study reports the first available complete genomes of this virus in Brazil and South America and contributes to understanding the etiological, epidemiological, evolutionary and disease-preventive knowledge about REV and its role in diseases caused by multiple viral coinfections.

## 2. Materials and Methods

### 2.1. Clinical Cases and Samples

A retrospective survey included poultry cases that previously tested positive for REV based on PCR targeting the Long Terminal Repeats (unpublished data). These cases included a total of 40 samples collected from 10 poultry farm cases (broilers and layer hens, Table S2) with pathological findings including proventriculitis, gizzard erosion, stunted growth, cachexia, and increased mortality (Table S2). Basically, the vaccination program included MDV, infectious bursal disease virus (IBDV), infectious bronchitis virus (IBV), and Newcastle disease virus (NDV). Each sample consisted of a pool from a specific organ that had been collected from 5 different birds from the same flock. These specific organs included: proventriculus, gizzard, thymus, bursa, intestine, liver and spleen. These samples were stored in the Laboratory of Avian Diseases at the School of Veterinary Medicine (University of São Paulo) from 2014 to 2019 at −20 °C until processing. Samples were homogenized in sterile phosphate-buffered saline (PBS), pH 7.4, in a 1:1 ratio for a total volume of 1.5 mL. Then they were frozen (−80 °C for 10 min) and thawed (56 °C for 1 min) three times [17]. Finally, the samples were centrifuged for 20 min at 12,000× *g*, and the supernatants were stored at −80 °C for subsequent procedures. This study was approved by the Ethics Commission on Animal Use of the School of Veterinary Medicine, University of São Paulo (FMVZUSP), under CEUAVET protocol no. 1727010620.

### 2.2. Detection of REV and Concomitant Viruses

A volume of 200 μL of the supernatants was used for simultaneous extraction of nucleic acids with the MagMAX^TM^ Viral/Pathogen Nucleic Acid Isolation Kit (Applied Biosystems, Austin, TX, USA). DNA and RNA concentration and quality were evaluated in a NanoDrop 2000 (Thermo Fisher Scientific, Wilmington, DE, USA). Reverse transcription reactions were performed to test RNA viruses with M-MLV Reverse Transcriptase (Invitrogen^TM^, Carlsbad, CA, USA) according to the manufacturer’s recommendations.

Nucleic acids were tested for the presence of REV by qPCR assays, targeting the long terminal repeat regions (LTRs), as well as *gag*, *pol*, and *env* genes [18,19,20,21]. Then, concomitant viral agents potentially associated with the observed clinical signs (proventriculitis, gizzard erosion, stunted growth, cachexia, and increased mortality without respiratory, cutaneous, or neoplastic signs) were also tested by qPCR. These viruses included avian nephritis virus (ANV), avian orthoreovirus (ARV), avian rotavirus A (ARtV-A), chicken anemia virus (CAV), chicken astrovirus (CAstV), chicken parvovirus (ChPV), chicken proventricular necrosis virus (CPNV) and fowl adenovirus (FAdV) (Table 1). All qPCR tests were performed with PowerUp^TM^ SYBR^TM^Green Master Mix (Applied Biosystems, Austin, TX, USA) with previously reported primers (Table 1).

Positive controls for REV, ARV, ARtV-A, ANV, CAstV, ChPV and FAdV were obtained from previous studies [14,15,23,24,29,30]. A positive control for CAV was obtained from a commercial vaccine (Circomune^®^, Ceva Animal Health Ltd., Amersham, UK). A positive control for CPNV was synthetized as a DNA fragment (Invitrogen^TM^ GeneArt^TM^ Strings^TM^) according to the R11/3 isolate sequence (HM038436). Sterile PBS was used as a negative control.

### 2.3. Genome Sequencing

Available REV genome sequences (Table S1) were retrieved from GenBank and aligned with an iterative refinement method (FFT-NS-i) on MAFFT v7.489 [31] to select conserved motifs shared by all the genomes. Complete pro-viral genome sequencing of REV was performed by amplifying thirteen overlapping regions (Table 2). The prediction of secondary structures (hairpins, self-dimers and heterodimers) in designed primers was evaluated with the OligoAnalyzer tool from the IDT website (https://www.idtdna.com).

PCR amplification was performed with 100 ng of DNA template in a reaction mix with final concentrations of 0.2 mM of each dNTP, 2 mM of MgCl_2_, 0.6 μM of each primer, and 0.75 U of Platinum^TM^ Taq DNA Polymerase with 1X PCR buffer (Thermo Fisher Scientific Baltics UAB, Vilnius, Lithuania). Thermal conditions started with an initial denaturation step at 94 °C for 3 min, followed by 36 cycles at 94 °C for 45 s, 60 °C for 45 s, and 72 °C for 90 s and a final elongation step at 72 °C for 10 min. These conditions were common for all regions, with the exception of LTR 5′ and LTR 3′ amplification, which were amplified at 56 °C for the annealing primer step.

LTR primers were designed to exclusively amplify the 5′ or 3′ regions. To sequence these regions completely, fragments of the expected size were purified from electrophoresis gels with the QIAquick^®^ Gel Extraction Kit (QIAGEN, Hilden, Germany). Amplified fragments were subsequently cloned according to previous procedures [32]. Briefly, we used the NEB^®^ PCR Cloning Kit and the pMiniT 2.0 vector with toxic minigene. Cloned vector-carrying bacteria were plated with ampicillin at 37 °C overnight for selection. Plasmids were purified with the PureLink^TM^ Quick Plasmid Miniprep Kit (Thermo Fisher Scientific Baltics UAB, Vilnius, Lithuania). All PCR products were purified with a QIAquick^®^ Gel Extraction Kit (QIAGEN, Hilden, Germany). Sequencing was performed on a 3500xL Genetic Analyzer with a BigDye^TM^ Terminator v3.1 Cycle Sequencing Kit (Applied Biosystems, Foster City, CA, USA) for all overlapping fragments, including the plasmids containing the 5′ and 3′ regions.

### 2.4. Sequence and Phylogenetic Analyses

Sequence products were trimmed, and the complete REV provirus genomes were de novo assembled with Geneious Prime^®^ 2020.2.4. (www.geneious.com). Phylogenetic analysis was conducted by comparing the complete genomes and the complete *gag*, *pol* and *env* gene sequences of Brazilian REVs with reference sequences obtained from the GenBank database. Multiple alignment was performed with an iterative refinement method (FFT-NS-i) on MAFFT v7.48 [31]. The best-fit substitution models for phylogenetic analysis were estimated with JModelTest 2 v2.1.10 [33], with the Akaike Information Criteria (AIC). The construction of phylogenetic trees was performed with MEGA X [34] with the maximum likelihood method and 1000 bootstrap replicates and edited with iTOL v6.4 [35]. Pairwise sequence identity scores and matrices for nucleotides and deduced amino acids were estimated with the SDT v1.2 tool [36].

### 2.5. Polymorphism Analysis and Protein Modeling

Analyses of polymorphism associated with *gag*, *pol* and *env* were conducted for amino acid changes in Geneious Prime^®^ 2020.2.4. (www.geneious.com). Two Brazilian strains corresponding to the samples sequenced in this study, along with two prototypes and/or subtype representative REV strains, were chosen for structural analysis and modeling of the *gag*, *pol* and *env* proteins. These were USP-586 (OK631657) and USP-976 (OK631658), HA9901 (NC_006934), SNV (DQ003591) and APC-566 (DQ387450).

To improve the modeling, we used the InterProScan tool [37] to explore functional sites, domain profiles, signatures, and protein families in the CDD, Pfam, PANTHER, SMART, PROSITE profiles and patterns, SUPERFAMILY, and CATH-Gene3D databases. In addition, MobiDBLite was included for intrinsic disorder prediction, TMHMM for transmembrane prediction and Coils for coiled-coil prediction for *env* sequences. These findings were compared to functional information about their closest relatives identified by BLAST against the UniProtKB database, with an E-threshold of 0.0001, and a BLOSUM62 matrix, for *gag* (avian spleen necrosis virus, UniProtID:P03342), *pol* (avian reticuloendotheliosis virus, UniProtID:P03360), and *env* (avian spleen necrosis virus, UniProtID:P31796 and avian reticuloendotheliosis virus, UniProtID:P03399).

Then, the selected sequences were aligned with the Clustal Omega tool and displayed in the MView alignment editor. For secondary structure prediction, *gag*, *pol*, and *env* sequences were sent to PRALINE, which used YASPIN for secondary prediction and the PHOBIUS method for transmembrane structure prediction. *Gag* and *pol* structures were built by I-TASSER, while the membrane *env* proteins structures were built by C-I-TASSER, since higher-accuracy contact maps have a significant influence on membrane structure modeling [38]. To validate the developed models, we considered the functional sites and secondary structure predictions, as well as the statistical and structural analyses.

The prediction of *N*-glycosylation was performed with NetNGlyc v1 [39]. *N*-acetylglucosamine posttranslational modification sites were built for *env* models using GlyProt on the GLYCOSCIENCES.de portal. In PyMOL (The PyMOL Molecular Graphics System, Version 2.0 Schrödinger, LLC.), we manually inserted the lipidation modification sites for *env* and *gag*, as well as one S-palmitoyl modification in CYS547 of the HA9901 (NC_006934) *env* model and CYS549 of the other *env* model, and one *N*-myristoyl glycine PTM for all *gag* models. All *gag* and *pol* models with less than 90% of the favored regions were calculated by completed structural refinement using locPREFMD. Protein superimpositions and visualizations were performed in Protein Imager.

We analyzed important *env* sequences, such as epitopes, motifs, and other sequences related to this protein. We compared *env* epitopes [40,41,42], fusion peptides, immunosuppression motifs and other based on the information from a reviewed UniProt sequence (UniProtID:P31796). A conservation analysis was performed with the same 38 complete *gag*, *pol* and *env* sequences.

## 3. Results

### 3.1. Detection of REV and Concomitant Viruses

REV was detected by qPCR in 65% of the tested samples (26/40). These included the samples from proventriculus (*n* = 6), liver (*n* = 6), spleen (*n* = 5), gizzard (*n* = 5), intestine (*n* = 2), and bursa (*n* = 2) (Table S2).

In the case of each farm (Table 3), considering as positive if at least one sample from that farm was positive for each specific virus, REV was detected in all of them (10/10). Coinfections with two or more viruses were detected in 90% (9/10) of farms, with 2 to 7 viruses detected. Solely infection with REV was detected in one farm (Farm 2, ID: 599). The coinfections were found as follows: 40% (4/10) of farms for ANV, 70% (7/10) of farms for ARV, 40% (4/10) of farms for CAV, 80% (8/10) of farms for CAstV, 90% (9/10) of farms for ChPV, 30% (3/10) of farms for FAdV. ARtV-A and CPNV were not detected (Table 3).

### 3.2. Genome Sequence and Phylogenetic Analyses

Complete sequencing was conducted in farm samples with sufficient genomic concentrations (USP-586 and USP-976). The USP-586 strain presented 8284 nucleotides of extension, with a 52.28% GC content. On the other hand, the USP-976 strain presented 8284 nucleotides and a 52.29% GC content. Complete genome sequences of the referred strains were submitted to GenBank under the accession numbers OK631657 (USP-586) and OK631658 (USP-976).

**Figure 1:**
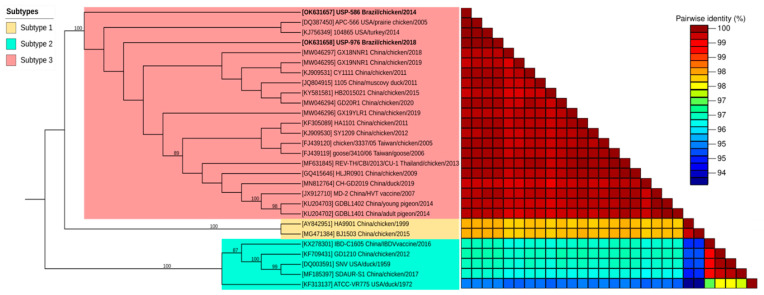
Phylogenetic analysis based on REV complete genomes. On the left: evolutionary tree inferred with the maximum likelihood method with 1000 bootstrap replications. The evolutionary distances were computed using the GTR + I model. On the right: genomic pairwise identity was calculated with the SDT v1.2 program and was plotted as color ranges according to the percentages of nucleotide identity. Brazilian strains of this study are highlighted by bold text.

The best nucleotide substitution models for complete LTR, *gag*, *pol*, *env* and REV genomes were K80, HKY + I, HKY + I, HKY and GTR + I, respectively. Phylogenetic analysis based on the complete genomes of REV revealed a clustering of the Brazilian strains (USP-586 and USP-976) together with the strains of Subtype 3 (Figure 1). This classification was well supported by the bootstrap test with 100% of replications for each subtype cluster. Both Brazilian strains were closer to each other and closer to the USA strains APC-566 and 104865 detected in prairie chicken and turkey, respectively. This proximity was also observed in the nucleotide pairwise comparison of complete genomes in the identity matrix (Table S3). Nucleotide identity ranged from 99.7 to 99.997% against subtype 3, 98.1% against subtype 1, and 95.2 to 96.5% against subtype 2. The USP-586 strain was different from USP-976 in one single nucleotide (99.997% identity). Additionally, the most identical REV reference genome to USP-586 was that of the APC-566 strain (99.997% identity), and for USP-976, it was that of the 104865 strain (99.997% identity).

We also inferred comparisons for total LTRs and coding genes. The phylogenetic tree for LTR regions situated the USP Brazilian strains into REV subtype 3; without enough bootstrap support, subtypes 1 and 2 appeared different (Figure S1). However, nucleotide identity ranged from 99.4 to 100.0% against subtype 3, in contrast with the lower values of 97.7 to 97.9% against subtype 1, and 92.0 to 94.6% against subtype 2 (Table S4).

In the case of the *gag* gene, phylogenetic inference situated the USP Brazilian strains into subtype 3 (Figure S2). Additionally, nucleotide identity ranged from 99.7 to 100.0% against REV subtype 3, 98.1 to 98.3 against subtype 1, and 96.1 to 97.9% against subtype 2 (Table S5). In terms of deduced amino acids, the identity ranged from 99.0 to 100.0% against subtype 3 (Table S5), 98.0 to 98.2% against subtype 1, and 95.8 to 98.0% against subtype 2. Five of the seven most identical strains with respect to our Brazilian strains were previously detected in the USA (“IL-74”, “104865”, “APC-566”, “FWPV-SD15-670.2”, and “FWPV-MN00.2”). The other two were FWPV-associated strains from India and Australia (“Ind/Guj-2011”, and “FWPV-S”, respectively).

For the *pol* gene, the phylogenetic tree situated the USP Brazilian strains into subtype 3 again (Figure S3) with good bootstrap support branching for each subtype. Nucleotide identity ranged from 99.6 to 100.0% against subtype 3, 98.3 to 98.4% against subtype 1, and 96.1 to 99.7% against subtype 2 (Table S6). Deduced amino acids revealed identity ranges from 99.1 to 100.0% against REV subtype 3, 98.4 to 99.0% against subtype 1, and 97.4 to 99.6% against subtype 2. Ten strains were 100.0% identical, revealing a high degree of conservation of this extensive gene.

Finally, for the *env* gene, phylogenetic inference situated the USP Brazilian strains into subtype 3 again (Figure S4), with high bootstrap support for every subtype node. Additionally, nucleotide identity ranged from 99.7 to 99.9% against REV subtype 3, 97.5 to 97.6 against subtype 1, and 94.7 to 99.7% against subtype 2 (Table S7). In terms of deduced amino acids, identity ranged from 98.8 to 100.0% against subtype 3 (Table S7), 95.4 to 96.1% against subtype 1, and 94.4 to 99.3% against subtype 2. Five strains had 100.0% identity with our studied strains (“IL-74”, “104865”, “FWPV-SD15-670.2”, “Ind/Guj-2011”, and “FWPV-S”).

### 3.3. Polymorphism Analysis and Protein Modeling

**Figure 2:**
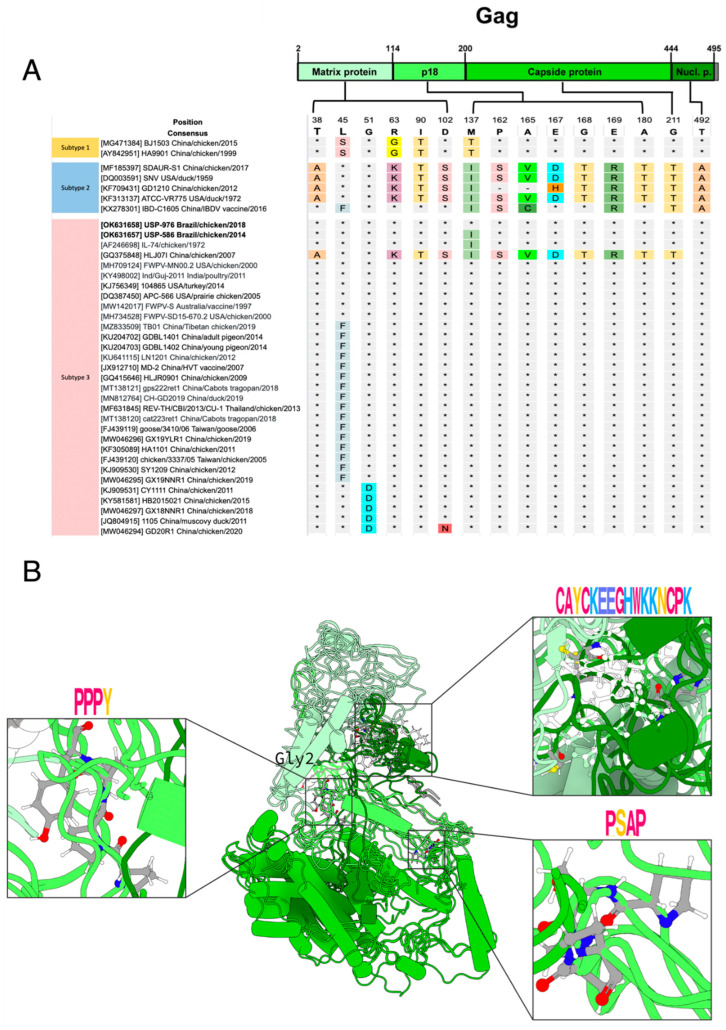
*Gag* protein polymorphism and modeling. (**A**) Localization of *gag* polymorphic sites toward their functional subdomains. The color scheme is the same used in the modeling. (**B**) Catalytic and motif consensus *gag* structure. On the left: the host interaction motif consensus PPXY. On the right: the zinc finger CCHC-type profile and the motif consensus PSAP. The motifs were plotted with WebLogo, and the *gag* structure was plotted with Protein Imager.

We compared the amino acid polymorphisms of our two Brazilian REV strains and representative complete coding sequences (CDS) against a panel of REV strains. The *gag* protein had 500 residues (positions in reference to NC_006934): the matrix protein (p12) from 2 to 114, p18 from 115 to 200, the capsid protein (p30) from 201 to 444 and the nucleocapsid protein (p10) from 445 to 495 [43] (Figure 2A). Regarding the variation in *gag* protein, we found 15 polymorphic sites (Figure 2). *Gag* is divided into four subdomains: the matrix protein (MA), p18, capsid protein (CA) and nucleocapsid protein (NC). No novel amino acid substitutions were identified in the four subdomains in our two Brazilian strains (USP-586 and USP-976). The strain HLJ071, which belongs to subtype 3, shares 12 polymorphisms with strains of subtype 2 (Figure 2). MA has six polymorphic sites (38, 45, 51, 63, 90 and 102). At site 45 of MA, polymorphic phenylalanine (F) appears in 17 Asian strains and different hosts; of these 17 strains, only the strain IBD-C1605 belongs to subtype 2, and the rest belong to subtype 3. In addition, site 51 of MA has polymorphic aspartic acid (D), which is shared by five Chinese strains from subtype 3. p18 is the subdomain with the most polymorphisms; it has seven polymorphic sites (162, 165, 167, 168, 169 and 180). We identified only one amino acid polymorphism in USP-586 (M137I) inside p18, which was shared with all strains of subtype 2 and with two strains of subtype 3 (IL-74 and HLJ071). We found the two non-polar motifs in the p18 of REV strains, PSAP (Figure 2B, bottom-right box), and PPPY (Figure 2B, left box) highly conserved. The last two subdomains CA and NU presented only one polymorphic site each, site 211 for CA and 492 for NU. We also found CCHC-type profile (Figure 2B, top-right box) in the NC of the REV strains. The predicted polymorphism of *N*-myristoylation at glycine in site 2 was found to be conserved across all sequences.

Three *gag* domains identified included the matrix protein (Pfam: PF01140, CATH-Gene3D: G3DSA:1.10.150.180, and SUPERFAMILY:SSF47836), capsid protein (Pfam: PF02093, and CATH-Gene3D: G3DSA:1.10.375.10), and nucleocapsid protein (CATH-Gene3D: G3DSA:4.10.60.10, SUPERFAMILY: SSF57756) with its zinc finger CCHC-type profile (SMART:SM00343, and PROSITE profiles: PS50158). The p18 domain, located between the first two domains, was not found in any database but was predicted to be a disordered region.

**Figure 3:**
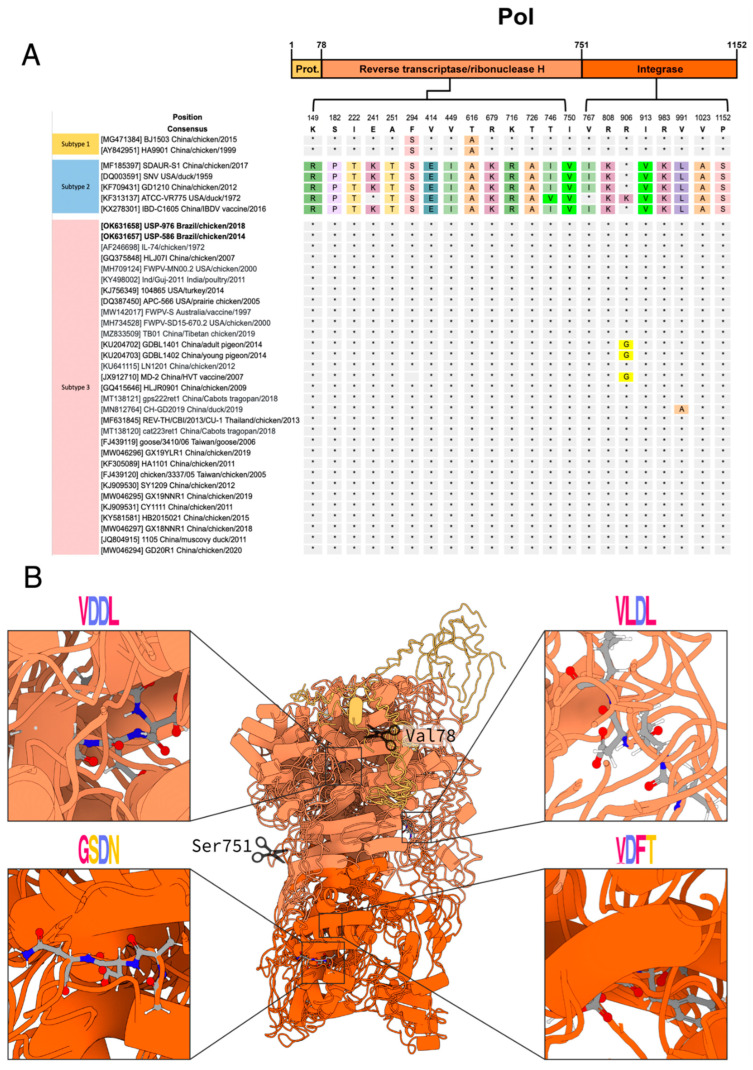
*Pol* protein polymorphism and modeling. (**A**) Localization of *pol* polymorphic sites toward their functional subdomains. The color scheme is the same used in the modeling. (**B**) Catalytic and motif consensus *pol* structure. On the top: the RT polymerase active sites that bind to Mg. On the bottom: integrase active sites that bind to Mn or Mg. The motifs were plotted with WebLogo, and the *pol* structure was plotted with Protein Imager.

The *pol* protein had 1152 residues (positions in reference to NC_006934): the protease from 1 to 78, the reverse transcriptase/ribonuclease H from 79 to 751 and the integrase from 752 to 1152 (Figure 3A). Regarding *pol* protein polymorphisms, we found 22 amino acid sites (Figure 3). No novel amino acid substitutions were identified in the Brazilian strains USP-586 and USP-976. The amino acid sequence of the retroviral protease (PR) lacked polymorphisms (Figure 3). On the other hand, reverse transcriptase (RT)/ribonuclease H had 14 polymorphic sites; however, the sequences were clearly conserved for each subtype, and only ATCC-VR775 diverged in two sites (241 and 746) from the other sequences of subtype 2. In addition, subtypes 1 and 2 shared the same polymorphism at sites 294 and 616. RT presented the active sites (Figure 3B, top-right and top-left boxes), highly conserved. Similar to the previous one, integrase (IN) presents homogeneous polymorphisms for each subtype, except for ATCC-VR775 in subtype 2, and GDBL1401, GDBL1402 and MD-2, which shared a particular polymorphism in site 906. IN presented a higher conservation of its catalytic residues (Figure 3B, bottom-right and bottom-left boxes).

*Pol* had two cleavage sites; the first “VT”, located at position 78–79, was conserved for all the analyzed sequences, and the second “SD” cleavage site, located at position 751-752, was also conserved for all sequences except for MF185397 and DQ003591, in which the amino acid serine was replaced by phenylalanine.

Regarding the five catalytic magnesium binding sites, the sites corresponding to reverse transcriptase/ribonuclease H, located at positions 228, 302 and 303, were conserved in all the analyzed sequences, and the amino acid was aspartic acid (D) for all three sites. The two catalytic sites located in the coding region for integrases, located at positions 874 and 933, were both conserved in all the sequences analyzed and corresponded to amino acid (D).

All modeled *pol* sequence alignment scores showed greater than 97 percent identity. The three domains found, in correlation with the literature, were the retroviral aspartyl protease (CDD: cd0695, Pfam: PF00077, CATH-Gene3D: G3DSA:2.40.70.10, SUPERFAMILY:SSF50630, and PROSITE Profiles: PS50175), the reverse transcriptase/ribonuclease H domain (CDD: cd03715, cd09273; Pfam: PF00078, PF17919, PF00075; CATH-Gene3D: G3DSA:3.30.70.270, G3DSA:3.30.420.10; SUPERFAMILY: SSF56672; and PROSITE Profiles: PS50878, PS50879), and the integrase domain (Pfam:PF09337, PF00665, PF18697; and PROSITE Profiles: PS50994).

**Figure 4:**
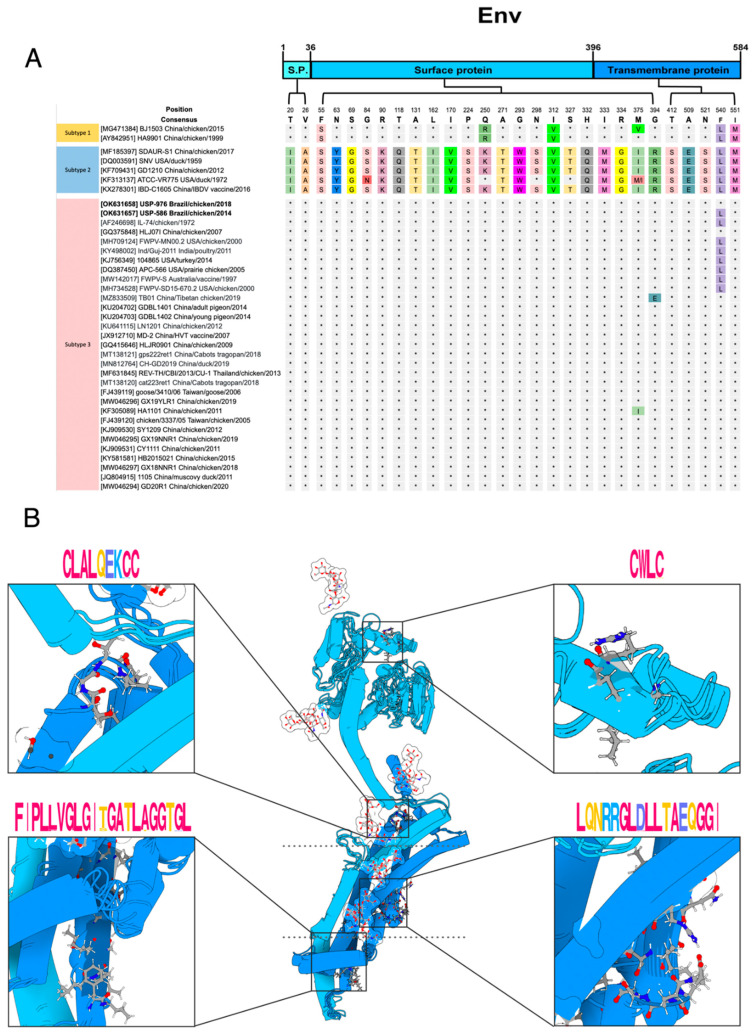
*Env* protein polymorphism and modeling. (**A**) Localization of *env* polymorphic sites toward their functional subdomains. The color scheme is the same used in the modeling. (**B**) Catalytic and motif consensus *env* structure. Top: the CXXC and CX6CC motifs that intervene in receptor recognition. Bottom: the fusion peptide and the immunosuppressive region, whose synthetic peptides inhibit immune function in vitro and in vivo. The motifs were plotted with WebLogo, and the *env* structure was plotted with Protein Imager.

The *env* protein has 584 residues (positions in reference to NC_006934). The signal peptide from 1 to 36, the surface protein (SU) from 37 to 396 and the transmembrane protein from 397 to 584 (Figure 4A). For the *env* protein variation, we found 29 polymorphic sites (Figure 4). The signal peptide has three polymorphic sites, the surface protein has 21 polymorphic sites and the transmembrane protein has five polymorphic sites. No novel amino acid substitutions were identified in the Brazilian strains USP-586 and USP-976.

Regarding the polymorphic sites in the *env* protein, subtypes 1 and 3 shared more residues in comparison to sequences of subtype 2. The REV sequences of subtype 1 have a serine in site 55, similar to the sequences of subtype 2. At site 250, the sequences of subtype 1 have arginine, almost all the sequences of subtype 2 have lysine, and the sequences of subtype 3 have glutamine. The sequences of subtypes 1 and 2 have valine at amino acid 312, while the sequences of subtype 3 have isoleucine. Subtypes 1 and 2 have a leucine residue at site 540, while most of the subtype 3 strains have phenylalanine. The strain USP-976 has the consensus residue of phenylalanine at site 540 similar to all sequences of subtype 3 from China, Thailand, and Taiwan. On the other hand, the strain USP-586 had a leucine in the same position as the sequences of subtype 3 from the USA, Australia, and India. The sequences of subtypes 1 and 2 have methionine in amino acid site 551, while the sequences of subtype 3 have isoleucine.

Xue et al. [41] reported the linear epitope ^213^SVQYHPL^219^, which is recognized by the neutralizing mAb A9E8, and was conserved in the 38 sequences analyzed for polymorphisms in the present study (Table S8). Cumberbatch et al. [40] identified five conserved peptides of REV *env* protein that can bind to a chicken’s MHC II, and Khairy et al. [42] identified two conserved epitopes that can be recognized by mAbs. These peptides are almost conserved in the 38 sequences analyzed; two of the peptides identified presented just one variation in the sequences of subtype 1 (Table S8).

Prediction of S-palmitoylation at cysteine 547 was found across all sequences. *N*-glycosylation was predicted at asparagine at eight sites (243, 272, 278, 304, 317, 326, 333, and 489) in almost all sequences. Site 272 presented the mutation N272E in subtype 1 strains BJ1503 (MG471384) and HA9901 (AY842951). Site 304 presented the mutation N304K in Chinese strain REV-Heilongjiang16 (MH186053).

The fusion peptide showed some variations (Figure 4B, bottom-left box). The changes L406P and A417T are presented in the two sequences of subtype 1 while the change T412S is presented in all sequences of subtype 2. The sequences of subtype 3, including the sequences of Brazilian strains USP-586 and USP-976, are well-conserved to the consensus sequence. The immunosuppression domain was well-conserved, and only one polymorphism in a sequence of subtype 3 was found (Asp469Gly) (Figure 4B, bottom-right box). The disulfide bond motifs present in SU and TM were well-conserved. There were only two variations, Cys258Ser in the ^255^CWLC^258^ motif (Figure 4B, top-right box), and Cys486Arg in the ^479^CLALQEKCC^487^ motif (Figure 4B, top-left box), both in sequences of subtype 3. The variations in the 38 sequences are shown in Table S8.

Almost all the modeled *env* sequences were identified as a viral envelope polyprotein (CDD: cd09851, Pfam: PF00429, CATH-Gene3D: G3DSA:1.10.287.210, SUPERFAMILY: SSF58069, and PANTHER: PTHR10424). The coiled-coil sites were predicted across the transmembrane region.

## 4. Discussion

REV is an avian pathogen associated with single or syndromic clinical manifestations that may include anemia, immunosuppression, proventriculitis, chronic neoplasia and runting disease. Since the first detection of REV-T in 1957, several species of domestic and wild birds have been described as susceptible to infection and disease development. However, complete REV genomes belonging to only few countries are currently available. Moreover, there are no complete genomes of Brazilian origin. Due to the aforementioned factors, we developed this study to characterize the complete genome of Brazilian REV strains detected in poultry farms with multiple viral coinfections.

We initially explored the presence of REV, and it was detected at a high frequency in the tested samples (65%). As these farms exhibited clinical signs affecting part of the gastrointestinal tract and low performance, we explored a possible coinfection with associated viral pathogens. Studies including REV coinfections are scarce and mostly limited to checking for oncogenic viruses [10,11] or pathologies with REV as an immunosuppressor agent [12,13]. In this study, the search for coinfections with viruses associated with enteric disorders and immunosuppression revealed simultaneous infections in most of the farms with up to seven of the tested viruses (including REV). We detected the presence of members of the families *Adenoviridae* (FAdV), *Anelloviridae* (CAV), *Astroviridae* (ANV and CAstV), *Parvoviridae* (ChPV), and *Reoviridae* (ARV).

The gastrointestinal tract of chickens is a complex environment and is commonly occupied with diverse microorganisms, including potential pathogenic viruses [30]. However, the specific contribution of each one to developing a disease is still unclear. ANV, ARV, CAstV, ChPV and FAdV are viruses associated to enteric disorders, growth suppression and even increased mortality, especially in young birds [22,23,26,27,28,29,30]. On the other hand, CAV is one of the most problematic immunosuppressor viruses associated with poor growth, anemia, cachexia and high mortality in broilers [44]. Most of our studied farms containing the major quantities of viral coinfections (ANV, ARV, CAV, CAstV, ChPV, FAdV, REV), were also exhibiting the worst clinical signs (proventriculitis, stunted growth, cachexia and mortality). In this scenario, the hypothesis of a single etiologic agent seems unlikely. However, multiple simultaneous viral infections could worsen the course of disease and even cause significant mortality [30]. Thus, the installed immunosuppression because of REV can trigger multiple coinfections and/or superinfections in the affected bird. Further, it is expected that infection with CAV and REV would exacerbate those pathologies [44].

Genome of Brazilian strains are in concordance with the genome size of subtype 3 strains of REV [43], which is shorter than the subtypes 1 and 2. Additionally, phylogenetic analysis and pairwise identity matrix based on complete genomes and complete LTR, *gag*, *pol* and *env* genes classified USP-586 and USP-976 into REV subtype 3. This finding agrees with those of previous studies performed with Brazilian partial sequences [14,15,16]. The origin and spread of REV strains around the world is very complex and uncertain. The USA strains were the most identical and could suggest the origin of Brazilian REV strains from the USA. These strains were detected between 1972 and 2015 and were isolated from different avian hosts (domestic chicken, prairie chicken, domestic turkeys, and wild turkeys). Surprisingly, most of these strains were also founded in coinfection or integrated into the genome of FWPV. There is much evidence about the association between FWPV and REV because of the etiologic origin of most referred REV strains [45]. The oldest of these originates from an FWPV strain, IL-74, isolated in Illinois, USA, in 1972 [46]. Subsequently, the integration of REV into the genome of that FWPV strain was confirmed [47]. The strain APC-566 was isolated from Attwater’s prairie chicken in Texas, USA, in 2005, and the authors reported an FWPV outbreak in that population the previous year [43]. Other USA strains, FWPV-MN00.2, isolated from chickens in Minnesota in 2000, and FWPV-SD15-670.2, isolated from Merriam’s wild turkeys in South Dakota in 2015, were reported to be integrated into the genome of FWPV [48]. The non-USA strains were Ind/Guj-2011, isolated from chickens in India in 2011, apparently within FWPV (GenBank No. KY498002), and the FWPV-S vaccine strain obtained in Australia in 1997 [49]. In fact, the association between FWPV and REV was proposed to have happened decades ago, prior to 1949 [50]. Since then, several reports have shown the coinfection and/or chimerization of both genomes [47,48], including in Brazil [15]. Based on this, an ancestral recombinant FWPV-REV may have given rise to the REV strains in the Americas. However, another source of infection that cannot be discarded is contamination and dispersion through vaccines, as already reported [8,19]. The origin of REV was proposed to be related to an ancient mammalian retrovirus, introduced into avian hosts, and then the generation of recombinant FWPV-REV [45]. Here, we report the relationship and identity clustering of North and South American REV strains as well as the probable association and coevolution of FWPV and REV in epidemiological and evolutionary terms involving the studied Brazilian strains.

Another objective of this study was to explore specific variability in proteins along the REV complete genome. MA, the second most polymorphic in REV *gag*, helps promote an interaction with the host’s plasma membrane [51]. The p18 was the most polymorphic *gag* protein. Even though the function of p18 inside REV is unknown so far, the PSAP motif increases budding efficiency, and the PPPY motif is central to the egress of the virus [52]. On the other hand, CA and NC possessed only 1 polymorphism in our study. CA encapsulates the genomic RNA and the replicative enzyme of the retrovirus [53]. NC has been reported to be efficiently promote the recognition of the genomic packaging signal and drive viral genomic RNA encapsulation specificity [54]. The CCHC-type profile in the NC of the REV strains is a zinc finger highly conserved inside the nucleocapsid protein of retroviruses, whose function is related to viral replication in the VIH retrovirus [55].

The high level of conservation of the *pol* gene may be associated with the relevant function of the proteins it encodes, as is the case of the PR which mediates proteolytic cleavages of *gag* and *gag*-*pol* polyproteins during or shortly after release of the virion from the plasma membrane [56,57]. In addition, RT is a multifunctional enzyme that converts viral RNA to double-stranded DNA in the cytoplasm [58,59], shortly after the virus enters the cell and the RH cleaves the RNA strand of the RNA-DNA heteroduplex. The RT enzyme has an active site that binds to two magnesium ions [60]. Additionally, IN is responsible for integrating linear reverse transcribed viral DNA into a chromosome of the infected host cell [61], its activity depends on magnesium or manganese ions and the higher conservation of its catalytic residues.

The *env* protein is an important glycoprotein that may have potential applications in the development of diagnostic techniques and epitope-based marker vaccines against REV [40,41]. The unique evaluated epitope for neutralization (^213^SVQYHPL^219^) of REV [41] was fully conserved in all the sequences analyzed. The other potential epitope peptides were well-conserved in all sequences. Although these epitopes have not been evaluated in immunization tests, the data available on their binding suggest that they would induce an immune response. The fusion peptide is localized in TM and is involved in fusion to the host cell plasma membrane [62]. The variations found here could provide valuable information about which subtype could best perform the fusion.

The immunosuppressive domain, also localized in TM, is involved in inhibiting the immune response [63,64]. This domain was well-conserved in the analyzed sequences, as expected. The formation of the disulfide bond between motifs of SU and TM [2,64] is thought to occur upon receptor recognition to allow membrane fusion, and the mutations found here would impact the formation of this bond. On the other hand, the first 200–300 aa of the *Gammaretrovirus* SU are defined as the receptor binding domain (RBD) [2]. Mutations on its domain could affect the binding ability and the host range as in the case of feline leukemia virus (FeLV) [65]. Further studies should be conducted on the structure and domains of REV proteins to better understand their functions and mechanisms of infection as well as to analyze if the variations found here could affect the host range of REV.

## 5. Conclusions

REV is an immunosuppressive agent that has been gradually detected in commercial and wild birds. In many cases, the pathology is caused by coinfection or superinfection with potentially harmful agents. Therefore, this study focused on REV, which was detected in chickens with multiple viral coinfections. We present the genomic characterization of Brazilian strains and suggest a close relationship with FWPV as well as USA-originated strains. In addition, the search for mutations and polymorphisms of these strains confirmed the high degree of genetic and structural conservation among REV subtype 3 throughout the entire genome, including the most important motifs, with a few exceptions in the *env* protein of other strains (Table S8). Because most of the viruses associated with pathologies in the gastroenteric tract do not have a commercial vaccine, epidemiological vigilance and characterization of the pathogens involved are essential to avoid or control the outbreaks and detrimental consequences of these diseases in the poultry industry and the surrounding environment.

## Figures and Tables

**Table 1:** Primers used for the detection of REV and concomitant viruses.

Virus	Gene Target	Primer Name	Primer Sequence	Reference
REV	LTR	LTR-F	AGGCTCATAAACCATAAAAGGAAATGT	[18]
		LTR-R	CCTTTACAACCATTGGCTCAGTATG	
	*gag*	qR 5	GTTTTCTATACACACCAGCCTACCT	[19]
		qR 6	TCCTGACCTCCCGCCTACT	
	*pol*	pol F	CCCCATTCATGTCCAGCTAT	[20]
		pol R	AGGGAGGAGAGGAGTGTTCC	
	*env*	gp90F	AAGAATCTGTGCGTGAAAG	[21]
		gp90R	TAAGGACCTGGTGAGTAGC	
ANV	UTR	ANV F	ACGGCGAGTACCATCGAG	[22]
		ANV R	AATGAAAAGCCCACTTTCGG	
ARV	S4	qREO-S4-F	GCTTTTTGAGTCCTTGTGCAG	[23]
		qREO-S4-R	GGATGTGTTGCGGGAAACT	
ARtV-A	VP6	qRtVA-VP6-F	TTGGACCAGTATTTCCTGCTG	[24]
		qRtVA-VP6-R	TGGTATGAGCTGTTACCCTCAA	
CAV	VP2/VP3	CAV-F630	ACGCTAAGATCTGCAACTG	[25]
		CAV-R756	TTACCCTGTACTCGGAGG	
CAstV	ORF1b-ORF2	CAstV F	GCYGCTGCTGAAGAWATACAG	[26]
		CAstV R	CATCCCTCTACCAGATTTTCTGAAA	
ChPV	NS	PVA-F	GCAACTAACCTGACCGTGTG	[27]
		PVA-R	CCCGGATTCAGAACCAGTAT	
CPNV	VP1	qCPNV-F	CGTAGACCTCGTCCTTCTGCTTKG	This study
		qCPNV-R	GGGCGGTAACCATTCAGATACAYCC	
FAdV	52K	52K-fw	ATGGCKCAGATGGCYAAGG	[28]
		52K-rw	AGCGCCTGGGTCAAACCGA	

**Table 2:** Primers used for complete genome sequencing of REV.

Gene Target	Primer Name	Primer Sequence	Location ^A^	Length (bp)
LTR 5′	LTR5-F1	AATGTGGGAGGGAGCTCYG	1–744	744
	LTR5-R1	CAMCAACAATCAGAWAYCACAGA		
*gag*	Gag-F2	GAGGRTTTGGGAGGATCGGAGTG	642–1415	774
	Gag-R2	GATATGGAGGTGGAGRRGCTG		
	Gag-F3	TGTAAACCCACAGGACCCTC	1253–1992	740
	Gag-R3	CCCTGCCGAACCTCAGTTAT		
	Gag-F4	TGGGAYCCTAACACRGGGAGA	1868–2616	749
	Gag-R4	TTCCGTATRTTCCCAGTAGCC		
*pol*	Pol-F5	ACTCGCCCAGGAGAGTAGAG	2269–3087	819
	Pol-R5	GTGTTCCAGGGGGAGTGGAC		
	Pol-F6	AAGTACCGCCCTACCTGTGA	2959–3827	869
	Pol-R6	CTTCCTCTTTTTCRCCCCAC		
	Pol-F7	CGAAAACCAAAAGGCARGTGCG	3680–4586	907
	Pol-R7	GGRCGTGTAGAGTRGCGAAT		
	Pol-F8	ACAAAGGCCCTGGAATGGAG	4505–5415	911
	Pol-R8	AGGGCCTCACACAACTGCTG		
	Pol-F9	ATGRTAACAGCCAAAGGGGG	5198–6086	889
	Pol-R9	AGTTGCTGCRAGGGGTRAC		
*env*	Env-F10	ACTGTTCCAACCTGGTGAYCT	5785–6537	753
	Env-R10	AATCATGTCAGTGGGACCGC		
	Env-F11	CGTATGAAGAYGGGCCTAAT	6413–7167	755
	Env-R11	GGGGATAAACTGGACTGCYC		
	Env-F12	GTGCATACTGGCATCAATCG	7050–7752	703
	Env-R12	CCACATTCCCCACYGCTCTT		
LTR 3′	LTR3-F13	TATTGTTCCTGACCCTCGGC	7577–8295	719
	LTR3-R13	CCCCCAAATGTTGTACMGAART		
pMiniT 2.0 vector	Flank-F	ACCTGCCAACCAAAGCGAGAA	-	Insert ^B^ + 309
	Flank-R	TCAGGGTTATTGTCTCATGAG		

^A^ According to the REV reference strain HA9901 (NC_006934). ^B^ According to the pMiniT 2.0 vector map (https://www.neb.com/).

**Table 3:** Detection of REV and concomitant viral infections on the studied farms.

Case	ID	Year	State ^A^	REV	ANV	ARV	CAV	CAstV	ChPV	FAdV	Total ^B^
Farm 1	586	2014	PR	+	−	−	−	+	+	−	3
Farm 2	599	2015	PR	+	−	−	−	−	−	−	1
Farm 3	976	2018	SP	+	+	−	+	−	+	−	4
Farm 4	1005	2018	SP	+	+	+	+	+	+	+	7
Farm 5	1006	2018	SP	+	+	+	+	+	+	+	7
Farm 6	1007	2018	SP	+	+	+	+	+	+	+	7
Farm 7	1270	2019	PR	+	−	+	−	+	+	−	4
Farm 8	1313	2019	PR	+	−	+	−	+	+	−	4
Farm 9	1314	2019	PR	+	−	+	−	+	+	−	4
Farm 10	1315	2019	PR	+	−	+	−	+	+	−	4
Total ^C^				10	4	7	4	8	9	3	

^A^ PR = PARANÁ. SP = SÃO PAULO. ^B^ Total number of concomitant viral infections. ^C^ Total of positive farms for each virus infection.

## Data Availability

The complete genome sequence and associated datasets generated during this study were deposited in GenBank under the accession numbers OK631657 (USP-586) and OK631658 (USP-976). All the protein models generated in this study were available in a GitHub repository (https://github.com/ElizaAlfaro/modelsREV; accessed on 24 February 2022).

## Supplementary Materials

The following are available online at https://www.mdpi.com/article/10.3390/v14040798/s1, Table S1. Complete genome REV strains used in the present study; Table S2. Detection of REV and concomitant viral infections for each sample in the studied farms; Table S3. Pairwise identity matrix of nucleotides for complete genomes of the compared strains of REV; Table S4. Pairwise identity matrix of nucleotides for long terminal repeats (LTRs) of the compared strains of REV; Table S5. Pairwise identity matrix of nucleotides and deduced amino acids for the *gag* gene of the compared strains of REV; Table S6. Pairwise identity matrix of nucleotides and deduced amino acids for the *pol* gene of the compared strains of REV; Table S7. Pairwise identity matrix of nucleotides and deduced amino acids for the *env* gene of the compared strains of REV; Table S8. Polymorphisms in *env* gene motifs of the compared strains of REV; Figure S1. Phylogenetic analysis based on the REV LTR region; Figure S2. Phylogenetic analysis based on the REV *gag* gene; Figure S3. Phylogenetic analysis based on the REV *pol* gene; Figure S4. Phylogenetic analysis based on the REV *env* gene.
