# Supplementary material for: Complete Genome Characterization of Reticuloendotheliosis Virus Detected in Chickens with Multiple Viral Coinfections
Source: Viruses. 2022 Apr 13;14(4):798. doi: 10.3390/v14040798 (PMC9028558; doi:10.3390/v14040798)
Supplement: Supplementary file 1 [file viruses-14-00798-s001.zip › viruses-1634948-supplementary.pdf]

viruses

Supplementary data

Complete Genome Characterization of REV Detected in Chickens with Multiple Viral Coinfections

Ruy D. Chacón[1,2], Benjy Sedano-Herrera[3,4], Elizabeth Regina Alfaro-Espinoza[5], Wilma Ursula Quispe Rojas[6], Arturo Liñan-Torres[7], David De la Torre[1], Anderson Bento de Oliveira[1], Claudete S. Astolfi-Ferreira[1], and Antonio J. Piantino Ferreira[1,*]

[1] Department of Pathology, School of Veterinary Medicine, University of São Paulo, Av. Prof. Orlando M. Paiva, 87, CEP 05508-270, São Paulo, Brazil; ruychaconv@usp.br (R.D.C.); daviddelatorreduque@gmail.com (D.D.T.); aoliveira@usp.br (A.B.O.); csastolfi@gmail.com (C.S.A.-F.); ajpferr@usp.br (A.J.P.F.);
[2] Inter-units Program in Biotechnology, University of São Paulo, Av. Prof. Dr. Orlando M. Paiva, 87, CEP 05508-270, São Paulo, Brazil;
[3] Departamento de Ecología, Museo de Historia Natural de la Universidad Nacional Mayor de San Marcos, Lima, 15072, Perú; benjy.sedano@unmsm.edu.pe (B.S-H.);
[4] Asociación Peruana de Astrobiología-ASPAST, Lima, Perú;
[5] Programa de Pós-Graduação em Bioinformática, Instituto de Ciências Biológicas, Universidade Federal de Minas Gerais, Belo Horizonte, Brazil; elizaespinoza@ufmg.br (E.R.A-E.);
[6] Laboratory of Molecular Microbiology and Biotechnology, Faculty of Biological sciences, Universidad Nacional Mayor de San Marcos, Lima, Perú; wilma.quispe@unmsm.edu.pe (W.U.Q.R.);
[7] Instituto de Biotecnología. Universidad Nacional Autónoma de México. Av. Universidad 2001 Col. Chamilpa, Cuernavaca, Morelos, 62210, México; arturo.linan@ibt.unam.mx (A.L-T.);
* Correspondence: ajpferr@usp.br; Tel.: +55-11-3091-1352

Table S1. Complete genome REV strains used in the present study.

Strain	Isolation year	Source	Country	Accession N°
SNV	1959	Duck	USA	DQ003591
ATCC-VR775	1972	Duck	USA	KF313137
HA9901	1999	Chicken	China	AY842951
APC-566	2005	Prairie Chicken	USA	DQ387450
chicken/3337/05	2005	Chicken	Taiwan	FJ439120
goose/3410/06	2006	Goose	Taiwan	FJ439119
HLJ07I	2007	Chicken	China	GQ375848
MD-2	2007	HVT Vaccine	China	JX912710
HLJR0901	2009	Chicken	China	GQ415646
1105	2011	Muscovy Duck	China	JQ804915
HA1101	2011	Chicken	China	KF305089
CY1111	2011	Chicken	China	KJ909531
GD1210	2012	Chicken	China	KF709431
SY1209	2012	Chicken	China	KJ909530
LN1201	2012	Chicken	China	KU641115
REV-TH/CBI/2013/CU-1	2013	Chicken	Thailand	MF631845
USP-586	2014	Chicken	Brazil	OK631657
104865	2014	Turkey	USA	KJ756349
GDBL1401	2014	Pigeon	China	KU204702
GDBL1402	2014	Pigeon	China	KU204703
HB2015021	2015	Chicken	China	KY581581
BJ1503	2015	Chicken	China	MG471384
IBD-C1605	2016	IBDV Vaccine	China	KX278301
SDAUR-S1	2017	Chicken	China	MF185397
USP-976	2018	Chicken	Brazil	OK631658
GX18NNR1	2018	Chicken	China	MW046297
GX19NNR1	2019	Chicken	China	MW046295
GX19YLR1	2019	Chicken	China	MW046296
CH-GD2019	2019	Duck	China	MN812764
TB01	2019	Tibetan Chicken	China	MZ833509
GD20R1	2020	Chicken	China	MW046294

viruses

MDPI

Table S2. Detection of REV and concomitant viral infections for each sample in the studied farms.

CASE	ID	YEAR	STATE	BIRDS	AGE[A]	SIGNS	SAMPLE[B]	REV	Coinfections
Farm 1	586	2014	PARANÁ	broilers	NI	proventriculitis, gizzard erosion	gizzard	**positive**	
							intestine	**positive**	
							liver	**positive**	
							proventriculus	**positive**	CAstV
							spleen	**positive**	ChPV
							thymus	negative	CAstV
Farm 2	599	2015	PARANÁ	broilers	NI	proventriculitis, gizzard erosion	gizzard	**positive**	
							intestine	**positive**	
							liver	**positive**	
							proventriculus	**positive**	
Farm 3	976	2018	SÃO PAULO	layer hens	40	proventriculitis, stunted growth, cachexia, mortality	bursa	negative	ANV, ChPV
							proventriculus	**positive**	ANV, ChPV
							thymus	negative	ANV, CAV, ChPV
Farm 4	1005	2018	SÃO PAULO	layer hens	38	proventriculitis, stunted growth, cachexia, mortality	bursa	**positive**	ANV, ARV, CAV, CAstV, ChPV, FAdV
							proventriculus	negative	ANV, ARV, CAV, CAstV, ChPV, FAdV
							thymus	negative	ANV, ARV, CAV, ChPV
Farm 5	1006	2018	SÃO PAULO	layer hens	32	proventriculitis, stunted growth, cachexia, mortality	bursa	**positive**	ANV, ARV, CAV, CAstV, ChPV, FAdV
							gizzard	negative	ANV, ARV, CAV, CAstV, ChPV
							proventriculus	negative	ANV, ARV, CAV, CAstV, ChPV
							thymus	negative	ANV, ARV, CAV, CAstV, ChPV
Farm 6	1007	2018	SÃO PAULO	layer hens	32	proventriculitis, stunted growth, cachexia, mortality	bursa	negative	ANV, ARV, CAV, CAstV, ChPV
							gizzard	**positive**	ANV, ARV, CAV, CAstV, ChPV, FAdV
							proventriculus	**positive**	ANV, ARV, CAV, CAstV, ChPV, FAdV
							thymus	negative	ANV, ARV, CAV, CAstV
Farm 7	1270	2019	PARANÁ	broilers	22	proventriculitis, gizzard erosion	gizzard	**positive**	ARV, CAstV, ChPV
							liver	**positive**	ChPV
							proventriculus	**positive**	ARV, CAstV, ChPV
							spleen	**positive**	ChPV

Farm 8	1313	2019	PARANÁ	broilers	22	proventriculitis, gizzard erosion	gizzard	negative	ARV, CAstV, ChPV
							liver	**positive**	ARV, CAstV
							proventriculus	negative	ARV, CAstV, ChPV
							spleen	**positive**	ARV, CAstV
Farm 9	1314	2019	PARANÁ	broilers	22	proventriculitis, gizzard erosion	gizzard	negative	CAstV, ChPV
							liver	**positive**	ARV, ChPV
							proventriculus	negative	CAstV, ChPV
							spleen	**positive**	ARV, ChPV
Farm 10	1315	2019	PARANÁ	broilers	22	proventriculitis, gizzard erosion	gizzard	**positive**	ARV, CAstV, ChPV
							liver	**positive**	ARV, CAstV, ChPV
							proventriculus	**positive**	ARV, CAstV, ChPV
							spleen	**positive**	ARV, CAstV, ChPV

[A] = age in days.

[B] = pool from a specific organ that had been collected from 5 different birds from the same flock.

NI = not informed.

viruses

MDPI

Table S3. Pairwise identity matrix of nucleotides for complete genomes of the compared strains of REV.

	**[OK631657] USP-586**	[DQ387450] APC-566	[KJ756349] 104865	**[OK631658] USP-976**	[MW046297] GX18NNR1	[MW046295] GX19NNR1	[KJ909531] CY1111	[JQ804915] 1105	[KY581581] HB2015021	[MW046294] GD20R1	[MW046296] GX19YLR1	[KF305089] HA1101	[KJ909530] SY1209	[FJ439120] chicken/3337/05	[FJ439119] goose/3410/06	[MF631845] REV-TH/CBI/2013/CU-1	[GQ415646] HLJR0901	[MN812764] CH-GD2019	[JX912710] MD-2	[KU204703] GDBL1402	[KU204702] GDBL1401	[AY842951] HA9901	[MG471384] BJ1503	[KX278301] IBD-C1605	[KF709431] GD1210	[DQ003591] SNV	[MF185397] SDAUR-S1	[KF313137] ATCC-VR775
**[OK631657] USP-586 Brazil/chicken/2014**																												
[DQ387450] APC-566 USA/prairie chicken/2005	99.997																											
[KJ756349] 104865 USA/turkey/2014	99.996	99.996																										
**[OK631658] USP-976 Brazil/chicken/2018**	99.997	99.996	99.997																									
[MW046297] GX18NNR1 China/chicken/2019	99.8	99.8	99.8	99.8																								
[MW046295] GX19NNR1 China/chicken/2019	99.7	99.8	99.8	99.8	99.6																							
[KJ909531] CY1111 China/chicken/2011	99.9	99.9	99.9	99.9	99.7	99.8																						
[JQ804915] 1105 China/muscovy duck/2011	99.8	99.9	99.9	99.9	99.7	99.7	99.8																					
[KY581581] HB2015021 China/chicken/2015	99.7	99.8	99.8	99.8	99.6	99.6	99.7	99.7																				
[MW046294] GD20R1 China/chicken/2020	99.9	99.9	99.9	99.9	99.7	99.7	99.9	99.8	99.7																			
[MW046296] GX19YLR1 China/chicken/2019	99.8	99.8	99.8	99.8	99.7	99.8	99.8	99.8	99.7	99.8																		
[KF305089] HA1101 China/chicken/2011	99.9	99.9	99.9	99.9	99.7	99.8	99.8	99.8	99.7	99.8	99.9																	
[KJ909530] SY1209 China/chicken/2012	99.8	99.8	99.9	99.9	99.7	99.7	99.8	99.8	99.7	99.8	99.8	99.9																
[FJ439120] chicken/3337/05 Taiwan/chicken/2005	99.9	99.9	99.9	99.9	99.7	99.7	99.8	99.8	99.7	99.8	99.8	99.9	99.8															
[FJ439119] goose/3410/06 Taiwan/goose/2006	99.9	99.9	99.9	99.9	99.7	99.8	99.8	99.8	99.7	99.8	99.9	99.9	99.9	99.9														
[MF631845] REV-TH/CBI/2013/CU-1 Thailand/chicken/2013	99.9	99.9	99.9	99.9	99.8	99.8	99.9	99.9	99.7	99.8	99.9	100.0	99.9	99.9	99.998													
[GQ415646] HLJR0901 China/chicken/2009	99.9	99.9	99.9	99.9	99.7	99.8	99.8	99.8	99.7	99.8	99.9	99.9	99.9	99.9	99.9	99.997												
[MN812764] CH-GD2019 China/duck/2019	99.7	99.7	99.7	99.7	99.6	99.6	99.7	99.7	99.5	99.6	99.7	99.8	99.7	99.7	99.8	99.8	99.8											
[JX912710] MD-2 China/HVT vaccine/2007	99.8	99.8	99.9	99.9	99.7	99.7	99.8	99.8	99.7	99.8	99.8	99.9	99.8	99.8	99.9	99.9	99.9	99.7										
[KU204703] GDBL1402 China/young pigeon/2014	99.8	99.8	99.8	99.8	99.7	99.7	99.7	99.7	99.6	99.7	99.8	99.8	99.8	99.8	99.8	99.9	99.9	99.7	99.997									
[KU204702] GDBL1401 China/adult pigeon/2014	99.8	99.8	99.8	99.8	99.6	99.6	99.7	99.7	99.6	99.7	99.7	99.8	99.7	99.8	99.8	99.9	99.8	99.7	99.9	99.998								
[AY842951] HA9901 China/chicken/1999	98.1	98.1	98.1	98.0	97.9	97.8	98.0	97.9	97.8	98.0	97.9	98.0	97.9	97.9	98.0	98.0	98.0	97.8	98.0	97.9	97.9							
[MG471384] BJ1503 China/chicken/2015	98.1	98.1	98.1	98.1	97.9	97.9	98.0	98.0	97.9	98.0	98.0	98.0	98.0	98.0	98.0	98.1	98.0	97.9	98.0	98.0	98.0	99.7						
[KX278301] IBD-C1605 China/IBDV vaccine/2016	96.4	96.3	96.4	96.3	96.2	96.2	96.3	96.3	96.1	96.3	96.2	96.3	96.3	96.3	96.3	96.3	96.3	96.2	96.3	96.3	96.2	94.6	94.7					
[KF709431] GD1210 China/chicken/2012	96.5	96.5	96.5	96.5	96.3	96.3	96.4	96.4	96.3	96.4	96.3	96.4	96.3	96.4	96.4	96.4	96.4	96.2	96.4	96.3	96.3	94.8	94.9	99.3				
[DQ003591] SNV USA/duck/1959	96.4	96.4	96.4	96.4	96.2	96.2	96.3	96.3	96.2	96.3	96.2	96.3	96.3	96.3	96.3	96.3	96.3	96.2	96.3	96.2	96.2	94.8	94.8	99.2	99.9			
[MF185397] SDAUR-S1 China/chicken/2017	96.2	96.2	96.2	96.2	96.1	96.0	96.1	96.1	96.0	96.2	96.1	96.2	96.1	96.1	96.2	96.2	96.2	96.0	96.1	96.1	96.1	94.6	94.6	99.0	99.7	99.8		
[KF313137] ATCC-VR775 USA/duck/1972	95.2	95.2	95.2	95.1	95.0	95.0	95.1	95.0	95.0	95.1	95.0	95.1	95.0	95.1	95.1	95.1	95.1	94.9	95.0	95.0	95.0	93.6	93.6	97.0	97.6	97.5	97.4	

Table S4. Pairwise identity matrix of nucleotides for long terminal repeats (LTRs) of the compared strains of REV.

	[MG471384] BJ1503	[AY842951] HA9901	[KJ756349] 104865	[DQ387450] APC-566	**[OK631658] USP-976**	[KU204702] GDBL1401	[KU204703] GDBL1402	[JX912710] MD-2	[GQ415646] HLJR0901	[MT138121] gps222ret1	[MN812764] CH-GD2019	[MF631845] REV-TH/CBI/2013/CU-1	[MT138120] cat223ret1	[FJ439119] goose/3410/06	[KJ909530] SY1209	[MW046296] GX19YLR1	[KF305089] HA1101	[FJ439120] chicken/3337/05	[MW046295] GX19NNR1	[KJ909531] CY1111	[KY581581] HB2015021	[MW046297] GX18NNR1	[JQ804915] 1105	[MW046294] GD20R1	**[OK631657] USP-586**	[MF185397] SDAUR-S1	[DQ003591] SNV	[KF709431] GD1210	[KF313137] ATCC-VR775	[KX278301] IBD-C1605
[MG471384] BJ1503 China/chicken/2015																														
[AY842951] HA9901 China/chicken/1999	99.8																													
[KJ756349] 104865 USA/turkey/2014	97.7	97.9																												
[DQ387450] APC-566 USA/prairie chicken/2005	97.7	97.9	99.9																											
**[OK631658] USP-976 Brazil/chicken/2018**	97.7	97.9	99.9	99.9																										
[KU204702] GDBL1401 China/adult pigeon/2014	97.5	97.7	99.4	99.4	99.4																									
[KU204703] GDBL1402 China/young pigeon/2014	97.5	97.7	99.4	99.4	99.4	99.9																								
[JX912710] MD-2 China/HVT vaccine/2007	97.7	97.9	99.6	99.6	99.6	99.8	99.8																							
[GQ415646] HLJR0901 China/chicken/2009	97.7	97.9	99.9	99.9	99.9	99.4	99.4	99.6																						
[MT138121] gps222ret1 China/Cabots tragopan/2018	97.7	97.9	99.9	99.9	99.9	99.4	99.4	99.6	99.9																					
[MN812764] CH-GD2019 China/duck/2019	97.7	97.9	99.9	99.9	99.9	99.4	99.4	99.6	99.9	99.9																				
[MF631845] REV-TH/CBI/2013/CU-1 Thailand/chicken/2013	97.7	97.9	99.9	99.9	99.9	99.4	99.4	99.6	99.9	99.9	99.9																			
[MT138120] cat223ret1 China/Cabots tragopan/2018	97.5	97.7	99.8	99.8	99.8	99.6	99.6	99.8	99.8	99.8	99.8	99.8																		
[FJ439119] goose/3410/06 Taiwan/goose/2006	97.7	97.9	99.9	99.9	99.9	99.4	99.4	99.6	99.9	99.9	99.9	99.9	99.8																	
[KJ909530] SY1209 China/chicken/2012	97.7	97.9	99.9	99.9	99.9	99.4	99.4	99.6	99.9	99.9	99.9	99.9	99.8	99.9																
[MW046296] GX19YLR1 China/chicken/2019	97.5	97.7	99.8	99.8	99.8	99.3	99.3	99.4	99.8	99.8	99.8	99.8	99.6	99.8	99.8															
[KF305089] HA1101 China/chicken/2011	97.7	97.9	99.9	99.9	99.9	99.4	99.4	99.6	99.9	99.9	99.9	99.9	99.8	99.9	99.9	99.8														
[FJ439120] chicken/3337/05 Taiwan/chicken/2005	97.7	97.9	99.9	99.9	99.9	99.4	99.4	99.6	99.9	99.9	99.9	99.9	99.8	99.9	99.9	99.8	99.9													
[MW046295] GX19NNR1 China/chicken/2019	97.7	97.9	99.9	99.9	99.9	99.4	99.4	99.6	99.9	99.9	99.9	99.9	99.8	99.9	99.9	99.8	99.9	99.9												
[KJ909531] CY1111 China/chicken/2011	97.7	97.9	99.9	99.9	99.9	99.4	99.4	99.6	99.9	99.9	99.9	99.9	99.8	99.9	99.9	99.8	99.9	99.9	99.9											
[KY581581] HB2015021 China/chicken/2015	97.7	97.9	99.9	99.9	99.9	99.4	99.4	99.6	99.9	99.9	99.9	99.9	99.8	99.9	99.9	99.8	99.9	99.9	99.9	99.9										
[MW046297] GX18NNR1 China/chicken/2019	97.5	97.7	99.8	99.8	99.8	99.3	99.3	99.4	99.8	99.8	99.8	99.8	99.6	99.8	99.8	99.6	99.8	99.8	99.8	99.8	99.8									
[JQ804915] 1105 China/muscovy duck/2011	97.7	97.9	99.9	99.9	99.9	99.4	99.4	99.6	99.9	99.9	99.9	99.9	99.8	99.9	99.9	99.8	99.9	99.9	99.9	99.9	99.9	99.8								
[MW046294] GD20R1 China/chicken/2020	97.7	97.9	99.9	99.9	99.9	99.4	99.4	99.6	99.9	99.9	99.9	99.9	99.8	99.9	99.9	99.8	99.9	99.9	99.9	99.9	99.9	99.8	99.9							
**[OK631657] USP-586 Brazil/chicken/2014**	97.7	97.9	99.9	99.9	99.9	99.4	99.4	99.6	99.9	99.9	99.9	99.9	99.8	99.9	99.9	99.8	99.9	99.9	99.9	99.9	99.9	99.8	99.9	99.9						
[MF185397] SDAUR-S1 China/chicken/2017	91.9	92.1	94.4	94.4	94.4	93.8	93.8	94	94.4	94.4	94.4	94.4	94.2	94.4	94.4	94.2	94.4	94.4	94.4	94.4	94.4	94.2	94.4	94.4	94.4					
[DQ003591] SNV USA/duck/1959	92.1	92.3	94.2	94.2	94.2	93.8	93.8	94	94.2	94.2	94.2	94.2	94.2	94.2	94.2	94	94.2	94.2	94.2	94.2	94.2	94	94.2	94.2	94.2	99.8				
[KF709431] GD1210 China/chicken/2012	92.4	92.6	94.6	94.6	94.6	94.2	94.2	94.4	94.6	94.6	94.6	94.6	94.6	94.6	94.6	94.4	94.6	94.6	94.6	94.6	94.6	94.4	94.6	94.6	94.6	99.8	99.8			
[KF313137] ATCC-VR775 USA/duck/1972	89.7	89.9	92	92	92	91.6	91.6	91.8	92	92	92	92	92	92	92	91.8	92	92	92	92	92	91.8	92	92	92	95.9	95.8	96.2		
[KX278301] IBD-C1605 China/IBDV vaccine/2016	92.1	92.3	94.6	94.6	94.6	94.2	94.2	94.4	94.6	94.6	94.6	94.6	94.6	94.6	94.6	94.4	94.6	94.6	94.6	94.6	94.6	94.4	94.6	94.6	94.6	99.8	99.5	99.8	96	

Table S5. Pairwise identity matrix of nucleotides[1] and deduced amino acids[2] for the *gag* gene of the compared strains of REV.

	[MG471384] BJ1503	[AY842951] HA9901	[MH709124] FWPV-MN00.2	[KY498002] Ind/Guj-2011	[KJ756349] 104865	[DQ387450] APC-566	**[OK631658] USP-976**	[MW142017] FWPV-S	[MH734528] FWPV-SD15-670.2	[MZ833509] TB01	[KU204702] GDBL1401	[KU204703] GDBL1402	[KU641115] LN1201	[JX912710] MD-2	[GQ415646] HLJR0901	[MT138121] gps222ret1	[MN812764] CH-GD2019	[MF631845] REV-TH/CBI/2013/CU-1	[MT138120] cat223ret1	[FJ439119] goose/3410/06	[KJ909530] SY1209	[MW046296] GX19YLR1	[KF305089] HA1101	[FJ439120] chicken/3337/05	[MW046295] GX19NNR1	[KJ909531] CY1111	[KY581581] HB2015021	[MW046297] GX18NNR1	[JQ804915] 1105	[MW046294] GD20R1	[AF246698] IL-74	**[OK631657] USP-586**	[MF185397] SDAUR-S1	[DQ003591] SNV	[KF709431] GD1210	[KF313137] ATCC-VR775	[GQ375848] HLJ07I	[KX278301] IBD-C1605
[MG471384] BJ1503 China/chicken/2015		99.8	98.2	98.2	98.2	98.2	98.2	98.2	98.2	98.0	98.2	98.2	98.2	98.2	98.2	98.2	97.6	98.2	97.8	98.2	97.6	98.2	98.0	98.2	97.8	97.6	98.0	97.6	97.6	97.8	98.2	98.2	96.0	96.4	96.7	95.2	96.6	94.4
[AY842951] HA9901 China/chicken/1999	99.7		98.0	98.0	98.0	98.0	98.0	98.0	98.0	97.8	98.0	98.0	98.0	98.0	98.0	98.0	97.4	98.0	97.6	98.0	97.4	98.0	97.8	98.0	97.6	97.4	97.8	97.4	97.4	97.6	98.0	98.0	95.8	96.2	96.5	95.0	96.4	94.2
[MH709124] FWPV-MN00.2 USA/chicken/2000	98.1	98.1		99.9	99.9	99.9	99.9	99.9	99.9	99.6	99.8	99.8	99.8	99.8	99.8	99.8	99.2	99.8	99.4	99.8	99.2	99.8	99.6	99.8	99.4	99.4	99.8	99.4	99.4	99.6	99.8	99.8	97.0	97.4	97.8	96.2	97.6	95.8
[KY498002] Ind/Guj-2011 India/poultry/2011	98.2	98.1	99.9		99.9	99.9	99.9	99.9	99.9	99.6	99.8	99.8	99.8	99.8	99.8	99.8	99.2	99.8	99.4	99.8	99.2	99.8	99.6	99.8	99.4	99.4	99.8	99.4	99.4	99.6	99.8	99.8	97.0	97.4	97.8	96.2	97.6	95.8
[KJ756349] 104865 USA/turkey/2014	98.2	98.1	99.9	99.9		99.9	99.9	99.9	99.9	99.6	99.8	99.8	99.8	99.8	99.8	99.8	99.2	99.8	99.4	99.8	99.2	99.8	99.6	99.8	99.4	99.4	99.8	99.4	99.4	99.6	99.8	99.8	97.0	97.4	97.8	96.2	97.6	95.8
[DQ387450] APC-566 USA/prairie chicken/2005	98.2	98.1	99.9	99.9	99.9		99.9	99.9	99.9	99.6	99.8	99.8	99.8	99.8	99.8	99.8	99.2	99.8	99.4	99.8	99.2	99.8	99.6	99.8	99.4	99.4	99.8	99.4	99.4	99.6	99.8	99.8	97.0	97.4	97.8	96.2	97.6	95.8
**[OK631658] USP-976 Brazil/chicken/2018**	98.2	98.1	99.9	99.9	99.9	99.9		99.9	99.9	99.6	99.8	99.8	99.8	99.8	99.8	99.8	99.2	99.8	99.4	99.8	99.2	99.8	99.6	99.8	99.4	99.4	99.8	99.4	99.4	99.6	99.8	99.8	97.0	97.4	97.8	96.2	97.6	95.8
[MW142017] FWPV-S Australia/vaccine/1997	98.2	98.1	99.9	99.9	99.9	99.9	99.9		99.9	99.6	99.8	99.8	99.8	99.8	99.8	99.8	99.2	99.8	99.4	99.8	99.2	99.8	99.6	99.8	99.4	99.4	99.8	99.4	99.4	99.6	99.8	99.8	97.0	97.4	97.8	96.2	97.6	95.8
[MH734528] FWPV-SD15-670.2 USA/Merriams wild turkey/2015	98.2	98.1	99.9	99.9	99.9	99.9	99.9	99.9		99.6	99.8	99.8	99.8	99.8	99.8	99.8	99.2	99.8	99.4	99.8	99.2	99.8	99.6	99.8	99.4	99.4	99.8	99.4	99.4	99.6	99.8	99.8	97.0	97.4	97.8	96.2	97.6	95.8
[MZ833509] TB01 China/Tibetan chicken/2019	98.0	97.9	99.7	99.8	99.8	99.8	99.8	99.8	99.8		99.8	99.8	99.8	99.8	99.8	99.8	99.2	99.8	99.4	99.8	99.2	99.8	99.6	99.8	99.4	99.0	99.4	99.0	99.0	99.2	99.4	99.4	96.6	97.0	97.4	95.8	97.2	95.8
[KU204702] GDBL1401 China/adult pigeon/2014	98.1	98.0	99.8	99.9	99.9	99.9	99.9	99.9	99.9	99.9		99.9	99.9	99.9	99.9	99.9	99.4	99.9	99.6	99.9	99.4	99.9	99.8	99.9	99.6	99.2	99.6	99.2	99.2	99.4	99.6	99.6	96.8	97.2	97.6	96.0	97.4	96.0
[KU204703] GDBL1402 China/young pigeon/2014	98.1	98.0	99.8	99.9	99.9	99.9	99.9	99.9	99.9	99.9	99.9		99.9	99.9	99.9	99.9	99.4	99.9	99.6	99.9	99.4	99.9	99.8	99.9	99.6	99.2	99.6	99.2	99.2	99.4	99.6	99.6	96.8	97.2	97.6	96.0	97.4	96.0
[KU641115] LN1201 China/chicken/2012	98.1	98.0	99.8	99.9	99.9	99.9	99.9	99.9	99.9	99.9	99.9	99.9		99.9	99.9	99.9	99.4	99.9	99.6	99.9	99.4	99.9	99.8	99.9	99.6	99.2	99.6	99.2	99.2	99.4	99.6	99.6	96.8	97.2	97.6	96.0	97.4	96.0
[JX912710] MD-2 China/HVT vaccine/2007	98.1	98.0	99.8	99.9	99.9	99.9	99.9	99.9	99.9	99.9	99.9	99.9	99.9		99.9	99.9	99.4	99.9	99.6	99.9	99.4	99.9	99.8	99.9	99.6	99.2	99.6	99.2	99.2	99.4	99.6	99.6	96.8	97.2	97.6	96.0	97.4	96.0
[GQ415646] HLJR0901 China/chicken/2009	98.1	98.0	99.8	99.9	99.9	99.9	99.9	99.9	99.9	99.9	99.9	99.9	99.9	99.9		99.9	99.4	99.9	99.6	99.9	99.4	99.9	99.8	99.9	99.6	99.2	99.6	99.2	99.2	99.4	99.6	99.6	96.8	97.2	97.6	96.0	97.4	96.0
[MT138121] gps222ret1 China/Cabots tragopan/2018	98.1	98.0	99.8	99.9	99.9	99.9	99.9	99.9	99.9	99.9	99.9	99.9	99.9	99.9	99.9		99.4	99.9	99.6	99.9	99.4	99.9	99.8	99.9	99.6	99.2	99.6	99.2	99.2	99.4	99.6	99.6	96.8	97.2	97.6	96.0	97.4	96.0
[MN812764] CH-GD2019 China/duck/2019	97.9	97.8	99.6	99.7	99.7	99.7	99.7	99.7	99.7	99.7	99.8	99.8	99.8	99.8	99.8	99.8		99.4	99.0	99.4	98.8	99.4	99.2	99.4	99.0	98.6	99.0	98.6	98.6	98.8	99.0	99.0	96.2	96.6	96.9	95.4	96.8	95.4
[MF631845] REV-TH/CBI/2013/CU-1 Thailand/chicken/2013	98.1	98.1	99.9	99.9	99.9	99.9	99.9	99.9	99.9	99.9	99.9	99.9	99.9	99.9	99.9	99.9	99.7		99.6	99.9	99.4	99.9	99.8	99.9	99.6	99.2	99.6	99.2	99.2	99.4	99.6	99.6	96.8	97.2	97.6	96.0	97.4	96.0
[MT138120] cat223ret1 China/Cabots tragopan/2018	98.0	97.9	99.7	99.8	99.8	99.8	99.8	99.8	99.8	99.7	99.8	99.8	99.8	99.8	99.8	99.8	99.6	99.9		99.6	99.0	99.6	99.4	99.6	99.2	98.8	99.2	98.8	98.8	99.0	99.2	99.2	96.4	96.8	97.1	95.6	97.0	95.6
[FJ439119] goose/3410/06 Taiwan/goose/2006	98.1	98.1	99.9	99.9	99.9	99.9	99.9	99.9	99.9	99.9	99.9	99.9	99.9	99.9	99.9	99.9	99.7	99.9	99.9		99.4	99.9	99.8	99.9	99.6	99.2	99.6	99.2	99.2	99.4	99.6	99.6	96.8	97.2	97.6	96.0	97.4	96.0
[KJ909530] SY1209 China/chicken/2012	97.9	97.9	99.7	99.7	99.7	99.7	99.7	99.7	99.7	99.7	99.7	99.7	99.7	99.7	99.7	99.7	99.5	99.8	99.7	99.8		99.4	99.2	99.4	99.0	98.6	99.0	98.6	98.6	98.8	99.0	99.0	96.2	96.6	96.9	95.4	96.8	95.4
[MW046296] GX19YLR1 China/chicken/2019	98.1	98.0	99.8	99.9	99.9	99.9	99.9	99.9	99.9	99.8	99.9	99.9	99.9	99.9	99.9	99.9	99.7	99.9	99.8	99.9	99.7		99.8	99.9	99.6	99.2	99.6	99.2	99.2	99.4	99.6	99.6	96.8	97.2	97.6	96.0	97.4	96.0
[KF305089] HA1101 China/chicken/2011	98.1	98.0	99.8	99.9	99.9	99.9	99.9	99.9	99.9	99.8	99.9	99.9	99.9	99.9	99.9	99.9	99.7	99.9	99.8	99.9	99.7	99.9		99.8	99.4	99.0	99.4	99.0	99.0	99.2	99.4	99.4	96.6	97.0	97.4	95.8	97.2	95.8
[FJ439120] chicken/3337/05 Taiwan/chicken/2005	98.1	98.0	99.8	99.9	99.9	99.9	99.9	99.9	99.9	99.8	99.9	99.9	99.9	99.9	99.9	99.9	99.7	99.9	99.8	99.9	99.7	99.9	99.9		99.6	99.2	99.6	99.2	99.2	99.4	99.6	99.6	96.8	97.2	97.6	96.0	97.4	96.0
[MW046295] GX19NNR1 China/chicken/2019	98.0	97.9	99.7	99.8	99.8	99.8	99.8	99.8	99.8	99.7	99.8	99.8	99.8	99.8	99.8	99.8	99.6	99.9	99.7	99.9	99.7	99.8	99.8	99.8		99.6	99.2	98.8	98.8	99.0	99.2	99.2	96.4	96.8	97.1	95.6	97.0	95.6
[KJ909531] CY1111 China/chicken/2011	97.9	97.9	99.7	99.7	99.7	99.7	99.7	99.7	99.7	99.5	99.6	99.6	99.6	99.6	99.6	99.6	99.4	99.7	99.5	99.7	99.5	99.6	99.6	99.6	99.8		99.6	99.2	99.2	99.4	99.2	99.2	96.4	96.8	97.1	95.6	97.0	95.2
[KY581581] HB2015021 China/chicken/2015	98.1	98.0	99.8	99.9	99.9	99.9	99.9	99.9	99.9	99.7	99.7	99.7	99.7	99.7	99.7	99.7	99.5	99.8	99.7	99.8	99.6	99.7	99.7	99.7	99.7	99.7		99.6	99.6	99.8	99.6	99.6	96.8	97.2	97.6	96.0	97.4	95.6
[MW046297] GX18NNR1 China/chicken/2019	97.8	97.7	99.5	99.6	99.6	99.6	99.6	99.6	99.6	99.4	99.5	99.5	99.5	99.5	99.5	99.5	99.3	99.5	99.4	99.5	99.3	99.5	99.5	99.5	99.4	99.5	99.6		99.2	99.4	99.2	99.2	96.4	96.8	97.1	95.6	97.0	95.2
[JQ804915] 1105 China/muscovy duck/2011	97.9	97.9	99.7	99.7	99.7	99.7	99.7	99.7	99.7	99.5	99.6	99.6	99.6	99.6	99.6	99.6	99.4	99.7	99.5	99.7	99.5	99.6	99.6	99.6	99.5	99.6	99.7	99.6		99.4	99.2	99.2	96.4	96.8	97.4	95.6	97.0	95.6
[MW046294] GD20R1 China/chicken/2020	98.1	98.1	99.7	99.8	99.8	99.8	99.8	99.8	99.8	99.6	99.7	99.7	99.7	99.7	99.7	99.7	99.5	99.7	99.6	99.7	99.5	99.7	99.7	99.7	99.6	99.7	99.8	99.5	99.7		99.4	99.4	96.8	97.2	97.6	96.0	97.4	95.4
[AF246698] IL-74/chicken/1972	98.3	98.2	99.9	99.9	99.9	99.9	99.9	99.9	99.9	99.7	99.8	99.8	99.8	99.8	99.8	99.8	99.6	99.9	99.7	99.9	99.7	99.8	99.8	99.8	99.7	99.7	99.8	99.5	99.7	99.7		99.9	97.2	97.6	98.0	96.4	97.8	96.0
**[OK631657] USP-586 Brazil/chicken/2014**	98.3	98.2	99.9	99.9	99.9	99.9	99.9	99.9	99.9	99.7	99.8	99.8	99.8	99.8	99.8	99.8	99.6	99.9	99.7	99.9	99.7	99.8	99.8	99.8	99.7	99.7	99.8	99.5	99.7	99.7	99.9		97.2	97.6	98.0	96.4	97.8	96.0
[MF185397] SDAUR-S1 China/chicken/2017	95.3	95.3	96.7	96.8	96.8	96.8	96.8	96.8	96.8	96.6	96.7	96.7	96.7	96.7	96.7	96.7	96.5	96.7	96.6	96.7	96.5	96.7	96.7	96.7	96.6	96.5	96.7	96.4	96.5	96.7	96.9	96.9		99.6	99.4	98.4	99.4	95.0
[DQ003591] SNV USA/duck/1959	95.5	95.5	96.9	97.0	97.0	97.0	97.0	97.0	97.0	96.8	96.9	96.9	96.9	96.9	96.9	96.9	96.7	96.9	96.8	96.9	96.7	96.9	96.9	96.9	96.8	96.7	96.9	96.6	96.7	96.9	97.1	97.1	99.8		99.8	98.8	99.8	95.4
[KF709431] GD1210 China/chicken/2012	95.7	95.7	97.2	97.2	97.2	97.2	97.2	97.2	97.2	97.0	97.1	97.1	97.1	97.1	97.1	97.1	96.9	97.2	97.0	97.2	97.0	97.1	97.1	97.1	97.0	97.0	97.1	96.8	97.0	97.2	97.3	97.3	99.8	99.9		98.6	99.6	96.3
[KF313137] ATCC-VR775 USA/duck/1972	94.5	94.6	96.0	96.1	96.1	96.1	96.1	96.1	96.1	95.9	95.9	95.9	95.9	95.9	95.9	95.9	95.7	96.0	95.9	96.0	95.8	95.9	95.9	96.1	95.9	95.8	95.9	95.7	95.8	96.0	96.1	96.1	98.0	98.2	98.2		98.6	94.2
[GQ375848] HLJ07I China/chicken/2007	96.4	96.3	97.9	97.9	97.9	97.9	97.9	97.9	97.9	97.7	97.8	97.8	97.8	97.8	97.8	97.8	97.6	97.9	97.7	97.9	97.7	97.8	97.8	97.8	97.7	97.7	97.8	97.5	97.7	97.9	98.0	98.0	98.7	98.9	98.9	97.5		95.2
[KX278301] IBD-C1605 China/IBDV vaccine/2016	94.6	94.7	96.2	96.3	96.3	96.3	96.3	96.3	96.3	96.4	96.4	96.4	96.4	96.4	96.4	96.4	96.2	96.4	96.2	96.4	96.2	96.3	96.3	96.3	96.2	96.0	96.2	95.9	96.2	96.1	96.4	96.4	97.1	97.3	97.8	95.8	96.2	

[1] Nucleotides comparison are below the diagonal line.

[2] Deduced amino acids comparison are above the diagonal line.

Table S6. Pairwise identity matrix of nucleotides[1] and deduced amino acids[2] for the *pol* gene of the compared strains of REV.

	[MG471384] BJ1503	[AY842951] HA9901	[MH709124] FWPV-MN00.2	[KY498002] Ind/Guj-2011	[KJ756349] 104865	[DQ387450] APC-566	**[OK631658] USP-976**	[MW142017] FWPV-S	[MH734528] FWPV-SD15-670.2	[MZ833509] TB01	[KU204702] GDBL1401	[KU204703] GDBL1402	[KU641115] LN1201	[JX912710] MD-2	[GQ415646] HLJR0901	[MT138121] gps222ret1	[MN812764] CH-GD2019	[MF631845] REV-TH/CBI/2013/CU-1	[MT138120] cat223ret1	[FJ439119] goose/3410/06	[KJ909530] SY1209	[MW046296] GX19YLR1	[KF305089] HA1101	[FJ439120] chicken/3337/05	[MW046295] GX19NNR1	[KJ909531] CY1111	[KY581581] HB2015021	[MW046297] GX18NNR1	[JQ804915] 1105	[MW046294] GD20R1	[AF246698] IL-74	**[OK631657] USP-586**	[MF185397] SDAUR-S1	[DQ003591] SNV	[KF709431] GD1210	[KF313137] ATCC-VR775	[GQ375848] HLJ07I	[KX278301] IBD-C1605
[MG471384] BJ1503 China/chicken/2015		99.5	98.9	99.0	99.0	99.0	99.0	99.0	99.0	98.1	98.7	98.9	99.0	98.9	99.0	98.9	98.7	99.0	98.9	98.9	98.9	98.8	99.0	98.9	98.5	98.9	98.4	98.8	98.8	98.9	98.9	99.0	96.7	97.1	97.4	97.0	98.5	97.4
[AY842951] HA9901 China/chicken/1999	99.7		98.4	98.4	98.4	98.4	98.4	98.4	98.4	97.6	98.2	98.4	98.4	98.4	98.4	98.4	98.2	98.4	98.4	98.4	98.4	98.3	98.4	98.4	98.0	98.4	97.9	98.3	98.3	98.4	98.4	98.4	96.2	96.6	96.9	96.4	98.0	96.9
[MH709124] FWPV-MN00.2 USA/chicken/2000	98.4	98.3		99.9	99.9	99.9	99.9	99.9	99.9	99.0	99.7	99.8	99.9	99.8	99.9	99.8	99.7	99.9	99.8	99.8	99.8	99.7	99.9	99.8	99.5	99.8	99.4	99.7	99.7	99.8	99.8	99.9	97.3	97.7	98.0	97.6	99.5	98.0
[KY498002] Ind/Guj-2011 India/poultry/2011	98.4	98.3	99.9		99.9	99.9	99.9	99.9	99.9	99.1	99.7	99.9	99.9	99.9	99.9	99.9	99.7	99.9	99.9	99.9	99.9	99.8	99.9	99.9	99.6	99.9	99.5	99.8	99.8	99.9	99.9	99.9	97.4	97.8	98.1	97.7	99.6	98.1
[KJ756349] 104865 USA/turkey/2014	98.4	98.3	99.9	99.9		99.9	99.9	99.9	99.9	99.1	99.7	99.9	99.9	99.9	99.9	99.9	99.7	99.9	99.9	99.9	99.9	99.8	99.9	99.9	99.6	99.9	99.5	99.8	99.8	99.9	99.9	99.9	97.4	97.8	98.1	97.7	99.6	98.1
[DQ387450] APC-566 USA/prairie chicken/2005	98.4	98.3	99.9	99.9	99.9		99.9	99.9	99.9	99.1	99.7	99.9	99.9	99.9	99.9	99.9	99.7	99.9	99.9	99.9	99.9	99.8	99.9	99.9	99.6	99.9	99.5	99.8	99.8	99.9	99.9	99.9	97.4	97.8	98.1	97.7	99.6	98.1
**[OK631658] USP-976 Brazil/chicken/2018**	98.4	98.3	99.9	99.9	99.9	99.9		99.9	99.9	99.1	99.7	99.9	99.9	99.9	99.9	99.9	99.7	99.9	99.9	99.9	99.9	99.8	99.9	99.9	99.6	99.9	99.5	99.8	99.8	99.9	99.9	99.9	97.4	97.8	98.1	97.7	99.6	98.1
[MW142017] FWPV-S Australia/vaccine/1997	98.4	98.3	99.9	99.9	99.9	99.9	99.9		99.9	99.1	99.7	99.9	99.9	99.9	99.9	99.9	99.7	99.9	99.9	99.9	99.9	99.8	99.9	99.9	99.6	99.9	99.5	99.8	99.8	99.9	99.9	99.9	97.4	97.8	98.1	97.7	99.6	98.1
[MH734528] FWPV-SD15-670.2 USA/Merriams wild turkey/2015	98.4	98.3	99.9	99.9	99.9	99.9	99.9	99.9		99.1	99.7	99.9	99.9	99.9	99.9	99.9	99.7	99.9	99.9	99.9	99.9	99.8	99.9	99.9	99.6	99.9	99.5	99.8	99.8	99.9	99.9	99.9	97.4	97.8	98.1	97.7	99.6	98.1
[MZ833509] TB01 China/Tibetan chicken/2019	98.1	97.9	99.6	99.6	99.6	99.6	99.6	99.6	99.6		98.9	99.0	99.1	99.0	99.1	99.0	98.9	99.1	99.0	99.0	99.0	99.0	99.1	99.0	98.7	99.0	98.6	99.0	99.0	99.0	99.0	99.1	96.5	97.0	97.2	96.8	98.7	97.2
[KU204702] GDBL1401 China/adult pigeon/2014	98.4	98.2	99.9	99.9	99.9	99.9	99.9	99.9	99.9	99.5		99.8	99.7	99.8	99.7	99.7	99.5	99.7	99.7	99.7	99.7	99.6	99.7	99.7	99.3	99.7	99.2	99.6	99.6	99.7	99.7	99.7	97.1	97.6	97.8	97.5	99.3	97.8
[KU204703] GDBL1402 China/young pigeon/2014	98.4	98.3	99.9	99.9	99.9	99.9	99.9	99.9	99.9	99.6	99.9		99.9	99.9	99.9	99.8	99.7	99.9	99.8	99.8	99.8	99.7	99.9	99.8	99.5	99.8	99.4	99.7	99.7	99.8	99.8	99.9	97.3	97.7	98.0	97.7	99.5	98.0
[KU641115] LN1201 China/chicken/2012	98.4	98.3	99.9	99.9	99.9	99.9	99.9	99.9	99.9	99.6	99.9	99.9		99.9	99.9	99.9	99.7	99.9	99.9	99.9	99.9	99.8	99.9	99.9	99.6	99.9	99.5	99.8	99.8	99.9	99.9	99.9	97.4	97.8	98.1	97.7	99.6	98.1
[JX912710] MD-2 China/HVT vaccine/2007	98.4	98.3	99.9	99.9	99.9	99.9	99.9	99.9	99.9	99.6	99.9	99.9	99.9		99.9	99.8	99.7	99.9	99.8	99.8	99.8	99.7	99.9	99.8	99.5	99.8	99.4	99.7	99.7	99.8	99.8	99.9	97.3	97.7	98.0	97.7	99.5	98.0
[GQ415646] HLJR0901 China/chicken/2009	98.4	98.3	99.9	99.9	99.9	99.9	99.9	99.9	99.9	99.6	99.9	99.9	99.9	99.9		99.9	99.7	99.9	99.9	99.9	99.9	99.8	99.9	99.9	99.6	99.9	99.5	99.8	99.8	99.9	99.9	99.9	97.4	97.8	98.1	97.7	99.6	98.1
[MT138121] gps222ret1 China/Cabots tragopan/2018	98.4	98.3	99.9	99.9	99.9	99.9	99.9	99.9	99.9	99.6	99.9	99.9	99.9	99.9	99.9		99.7	99.9	99.8	99.8	99.8	99.7	99.9	99.8	99.5	99.8	99.4	99.7	99.7	99.8	99.8	99.9	97.3	97.7	98.0	97.6	99.5	98.0
[MN812764] CH-GD2019 China/duck/2019	98.3	98.1	99.8	99.9	99.9	99.9	99.9	99.9	99.9	99.5	99.8	99.8	99.9	99.8	99.9	99.8		99.7	99.7	99.7	99.7	99.6	99.7	99.7	99.3	99.7	99.2	99.6	99.6	99.7	99.7	99.7	97.2	97.7	97.9	97.5	99.3	97.9
[MF631845] REV-TH/CBI/2013/CU-1 Thailand/chicken/2013	98.4	98.3	99.9	99.9	99.9	99.9	99.9	99.9	99.9	99.6	99.9	99.9	99.9	99.9	99.9	99.9	99.9		99.9	99.9	99.9	99.8	99.9	99.9	99.6	99.9	99.5	99.8	99.8	99.9	99.9	99.9	97.4	97.8	98.1	97.7	99.6	98.1
[MT138120] cat223ret1 China/Cabots tragopan/2018	98.4	98.2	99.9	99.9	99.9	99.9	99.9	99.9	99.9	99.6	99.9	99.9	99.9	99.9	99.9	99.9	99.8	99.9		99.8	99.8	99.7	99.9	99.8	99.5	99.8	99.4	99.7	99.7	99.8	99.8	99.9	97.3	97.7	98.0	97.6	99.5	98.0
[FJ439119] goose/3410/06 Taiwan/goose/2006	98.4	98.2	99.9	99.9	99.9	99.9	99.9	99.9	99.9	99.6	99.9	99.9	99.9	99.9	99.9	99.9	99.8	99.9	99.9		99.8	99.7	99.9	99.8	99.5	99.8	99.4	99.7	99.7	99.8	99.8	99.9	97.3	97.7	98.0	97.6	99.5	98.0
[KJ909530] SY1209 China/chicken/2012	98.4	98.2	99.9	99.9	99.9	99.9	99.9	99.9	99.9	99.5	99.8	99.9	99.9	99.9	99.9	99.9	99.8	99.9	99.9	99.9		99.7	99.9	99.8	99.5	99.8	99.4	99.7	99.7	99.8	99.8	99.9	97.3	97.7	98.0	97.6	99.5	98.0
[MW046296] GX19YLR1 China/chicken/2019	98.4	98.2	99.9	99.9	99.9	99.9	99.9	99.9	99.9	99.5	99.8	99.9	99.9	99.9	99.9	99.9	99.8	99.9	99.9	99.9	99.8		99.8	99.7	99.4	99.7	99.5	99.7	99.7	99.7	99.7	99.8	97.2	97.7	97.9	97.5	99.4	97.9
[KF305089] HA1101 China/chicken/2011	98.4	98.3	99.9	99.9	99.9	99.9	99.9	99.9	99.9	99.6	99.9	99.9	99.9	99.9	99.9	99.9	99.9	99.9	99.9	99.9	99.9	99.9		99.9	99.6	99.9	99.5	99.8	99.8	99.9	99.9	99.9	97.4	97.8	98.1	97.7	99.6	98.1
[FJ439120] chicken/3337/05 Taiwan/chicken/2005	98.4	98.2	99.9	99.9	99.9	99.9	99.9	99.9	99.9	99.5	99.8	99.9	99.9	99.9	99.9	99.9	99.8	99.9	99.9	99.9	99.8	99.8	99.9		99.5	99.8	99.4	99.7	99.7	99.8	99.8	99.9	97.3	97.7	98.0	97.6	99.5	98.0
[MW046295] GX19NNR1 China/chicken/2019	98.2	98.1	99.8	99.8	99.8	99.8	99.8	99.8	99.8	99.4	99.7	99.8	99.8	99.8	99.8	99.8	99.7	99.8	99.7	99.7	99.7	99.8	99.8	99.7		99.7	99.0	99.4	99.4	99.5	99.5	99.6	97.0	97.4	97.7	97.2	99.1	97.7
[KJ909531] CY1111 China/chicken/2011	98.4	98.3	99.9	99.9	99.9	99.9	99.9	99.9	99.9	99.6	99.9	99.9	99.9	99.9	99.9	99.9	99.8	99.9	99.9	99.9	99.9	99.9	99.9	99.9	99.8		99.4	99.7	99.7	99.8	99.8	99.9	97.4	97.7	98.0	97.6	99.5	98.0
[KY581581] HB2015021 China/chicken/2015	98.2	98.0	99.7	99.7	99.7	99.7	99.7	99.7	99.7	99.4	99.7	99.7	99.7	99.7	99.7	99.7	99.6	99.7	99.7	99.7	99.7	99.7	99.7	99.7	99.5	99.7		99.3	99.3	99.4	99.4	99.5	96.9	97.3	97.6	97.1	99.0	97.6
[MW046297] GX18NNR1 China/chicken/2018	98.3	98.1	99.8	99.9	99.9	99.9	99.9	99.9	99.9	99.5	99.8	99.8	99.9	99.8	99.9	99.8	99.7	99.9	99.8	99.8	99.8	99.8	99.9	99.8	99.7	99.8	99.6		99.7	99.7	99.7	99.8	97.2	97.7	97.9	97.5	99.4	97.9
[JQ804915] 1105 China/muscovy duck/2011	98.4	98.2	99.9	99.9	99.9	99.9	99.9	99.9	99.9	99.5	99.8	99.9	99.9	99.9	99.9	99.9	99.7	99.9	99.8	99.8	99.8	99.8	99.9	99.8	99.7	99.9	99.6	99.7		99.7	99.7	99.8	97.2	97.7	97.9	97.5	99.4	97.9
[MW046294] GD20R1 China/chicken/2020	98.4	98.2	99.9	99.9	99.9	99.9	99.9	99.9	99.9	99.5	99.8	99.9	99.9	99.9	99.9	99.9	99.8	99.9	99.9	99.9	99.8	99.9	99.9	99.8	99.8	99.9	99.7	99.8	99.8		99.8	99.9	97.3	97.7	98.0	97.6	99.5	98.0
[AF246698] IL-74/chicken/1972	98.4	98.3	99.9	99.9	99.9	99.9	99.9	99.9	99.9	99.6	99.9	99.9	99.9	99.9	99.9	99.9	99.8	99.9	99.9	99.9	99.9	99.9	99.9	99.9	99.8	99.9	99.7	99.8	99.9	99.9		99.9	97.3	97.7	98.0	97.6	99.7	98.0
**[OK631657] USP-586 Brazil/chicken/2014**	98.4	98.3	99.9	99.9	99.9	99.9	99.9	99.9	99.9	99.6	99.9	99.9	99.9	99.9	99.9	99.9	99.9	99.9	99.9	99.9	99.9	99.9	99.9	99.9	99.8	99.9	99.7	99.9	99.9	99.9	99.9		97.4	97.8	98.1	97.7	99.6	98.1
[MF185397] SDAUR-S1 China/chicken/2017	95.3	95.2	96.7	96.7	96.7	96.7	96.7	96.7	96.7	96.4	96.6	96.7	96.7	96.7	96.7	96.7	96.6	96.7	96.7	96.6	96.6	96.6	96.7	96.6	96.5	96.7	96.5	96.6	96.6	96.7	96.7	96.7		99.6	99.1	98.3	97.0	99.1
[DQ003591] SNV USA/duck/1959	95.5	95.3	96.8	96.8	96.8	96.8	96.8	96.8	96.8	96.5	96.8	96.8	96.8	96.8	96.8	96.8	96.7	96.8	96.8	96.8	96.8	96.8	96.8	96.8	96.6	96.8	96.6	96.7	96.7	96.8	96.8	96.8	99.9		99.6	98.7	97.4	99.6
[KF709431] GD1210 China/chicken/2012	95.6	95.4	96.9	97.0	97.0	97.0	97.0	97.0	97.0	96.6	96.9	96.9	97.0	96.9	97.0	96.9	96.8	97.0	96.9	96.9	96.9	96.9	97.0	96.9	96.8	96.9	96.8	96.8	96.8	96.9	96.9	97.0	99.7	99.9		99.0	97.7	99.8
[KF313137] ATCC-VR775 USA/duck/1972	94.9	94.7	96.1	96.1	96.1	96.1	96.1	96.1	96.1	95.7	96.0	96.1	96.1	96.1	96.1	96.1	96.0	96.1	96.1	96.0	96.0	96.0	96.1	96.1	95.9	96.1	95.9	96.0	96.0	96.0	96.1	96.1	97.9	98.1	98.2		97.2	99.0
[GQ375848] HLJ07I China/chicken/2007	98.1	98.0	99.7	99.7	99.7	99.7	99.7	99.7	99.7	99.3	99.6	99.7	99.7	99.7	99.7	99.7	99.6	99.7	99.7	99.7	99.6	99.6	99.7	99.6	99.5	99.7	99.5	99.6	99.6	99.6	99.7	99.7	96.5	96.6	96.7	95.9		97.7
[KX278301] IBD-C1605 China/IBDV vaccine/2016	95.5	95.4	96.9	96.9	96.9	96.9	96.9	96.9	96.9	96.6	96.8	96.9	96.9	96.9	96.9	96.9	96.8	96.9	96.9	96.8	96.8	96.8	96.9	96.8	96.7	96.9	96.7	96.8	96.8	96.9	96.9	96.9	99.7	99.8	99.9	98.1	96.7	

[1] Nucleotides comparison are below the diagonal line.

[2] Deduced amino acids comparison are above the diagonal line.

Table S7. Pairwise identity matrix of nucleotides[1] and deduced amino acids[2] for the *env* gene of the compared strains of REV.

	[MG471384] BJ1503	[AY842951] HA9901	[MH709124] FWPV-MN00.2	[KY498002] Ind/Guj-2011	[KJ756349] 104865	[DQ387450] APC-566	**[OK631658] USP-976**	[MW142017] FWPV-S	[MH734528] FWPV-SD15-670.2	[MZ833509] TB01	[KU204702] GDBL1401	[KU204703] GDBL1402	[KU641115] LN1201	[JX912710] MD-2	[GQ415646] HLJR0901	[MT138121] gps222ret1	[MN812764] CH-GD2019	[MF631845] REV-TH/CBI/2013/CU-1	[MT138120] cat223ret1
[MG471384] BJ1503 China/chicken/2015		99.7	95.5	95.7	95.7	95.5	95.4	95.7	95.7	94.7	95	95	95.5	95.4	95.5	95.5	95	95.5	95.2
[AY842951] HA9901 China/chicken/1999	99.8		95.9	96.1	96.1	95.9	95.7	96.1	96.1	95	95.4	95.4	95.9	95.7	95.9	95.9	95.4	95.9	95.5
[MH709124] FWPV-MN00.2 USA/chicken/2000	97.5	97.6		99.8	99.8	99.7	99.5	99.8	99.8	98.6	99.1	99.1	99.7	99.5	99.7	99.7	99.1	99.7	99.3
[KY498002] Ind/Guj-2011 India/poultry/2011	97.6	97.7	99.9		99.9	99.8	99.7	99.9	99.9	98.8	99.3	99.3	99.8	99.7	99.8	99.8	99.3	99.8	99.5
[KJ756349] 104865 USA/turkey/2014	97.6	97.7	99.9	99.9		99.8	99.7	99.9	99.9	98.8	99.3	99.3	99.8	99.7	99.8	99.8	99.3	99.8	99.5
[DQ387450] APC-566 USA/prairie chicken/2005	97.5	97.6	99.9	99.9	99.9		99.5	99.8	99.8	98.6	99.1	99.1	99.7	99.5	99.7	99.7	99.1	99.7	99.3
**[OK631658] USP-976 Brazil/chicken/2018**	97.5	97.5	99.8	99.9	99.9	99.8		99.7	99.7	98.8	99.3	99.3	99.8	99.7	99.8	99.8	99.3	99.8	99.5
[MW142017] FWPV-S Australia/vaccine/1997	97.6	97.7	99.9	99.9	99.9	99.9	99.9		99.9	98.8	99.3	99.3	99.8	99.7	99.8	99.8	99.3	99.8	99.5
[MH734528] FWPV-SD15-670.2 USA/Merriams wild turkey/2015	97.6	97.7	99.9	99.9	99.9	99.9	99.9	99.9		98.8	99.3	99.3	99.8	99.7	99.8	99.8	99.3	99.8	99.5
[MZ833509] TB01 China/Tibetan chicken/2019	97.1	97.2	99.4	99.4	99.4	99.4	99.4	99.4	99.4		98.5	98.5	99	98.8	99	99	98.5	99	98.6
[KU204702] GDBL1401 China/adult pigeon/2014	97.4	97.4	99.7	99.8	99.8	99.7	99.8	99.8	99.8	99.3		99.9	99.5	99.7	99.5	99.5	99	99.5	99.7
[KU204703] GDBL1402 China/young pigeon/2014	97.4	97.4	99.7	99.8	99.8	99.7	99.8	99.8	99.8	99.3	99.9		99.5	99.7	99.5	99.5	99	99.5	99.7
[KU641115] LN1201 China/chicken/2012	97.5	97.6	99.9	99.9	99.9	99.9	99.9	99.9	99.9	99.5	99.8	99.8		99.8	99.9	99.9	99.5	99.9	99.7
[JX912710] MD-2 China/HVT vaccine/2007	97.5	97.5	99.8	99.9	99.9	99.8	99.9	99.9	99.9	99.4	99.9	99.9	99.9		99.8	99.8	99.3	99.8	99.5
[GQ415646] HLJR0901 China/chicken/2009	97.4	97.5	99.8	99.8	99.8	99.8	99.8	99.8	99.8	99.4	99.7	99.7	99.9	99.8		99.9	99.5	99.9	99.7
[MT138121] gps222ret1 China/Cabots tragopan/2018	97.5	97.6	99.9	99.9	99.9	99.9	99.9	99.9	99.9	99.5	99.8	99.8	99.9	99.9	99.9		99.5	99.9	99.7
[MN812764] CH-GD2019 China/duck/2019	97.4	97.4	99.7	99.8	99.8	99.7	99.8	99.8	99.8	99.3	99.7	99.7	99.8	99.8	99.7	99.8		99.5	99.1
[MF631845] REV-TH/CBI/2013/CU-1 Thailand/chicken/2013	97.5	97.6	99.9	99.9	99.9	99.9	99.9	99.9	99.9	99.5	99.8	99.8	99.9	99.9	99.9	99.9	99.8		99.7
[MT138120] cat223ret1 China/Cabots tragopan/2018	97.4	97.5	99.8	99.8	99.8	99.8	99.8	99.8	99.8	99.4	99.9	99.9	99.9	99.8	99.8	99.9	99.7	99.9	
[FJ439119] goose/3410/06 Taiwan/goose/2006	97.5	97.5	99.8	99.9	99.9	99.8	99.9	99.9	99.9	99.4	99.8	99.8	99.9	99.9	99.8	99.9	99.8	99.9	99.8
[KJ909530] SY1209 China/chicken/2012	97.4	97.5	99.8	99.8	99.8	99.8	99.8	99.8	99.8	99.4	99.7	99.7	99.9	99.8	99.8	99.9	99.7	99.9	99.8
[MW046296] GX19YLR1 China/chicken/2019	97.4	97.5	99.8	99.8	99.8	99.8	99.8	99.8	99.8	99.4	99.7	99.7	99.9	99.8	99.8	99.9	99.7	99.9	99.8
[KF305089] HA1101 China/chicken/2011	97.4	97.5	99.8	99.8	99.8	99.8	99.8	99.8	99.8	99.4	99.7	99.7	99.9	99.8	99.8	99.9	99.7	99.9	99.8
[FJ439120] chicken/3337/05 Taiwan/chicken/2005	97.5	97.5	99.8	99.9	99.9	99.8	99.9	99.9	99.9	99.4	99.8	99.8	99.9	99.9	99.8	99.9	99.8	99.9	99.8
[MW046295] GX19NNR1 China/chicken/2019	97.3	97.4	99.7	99.7	99.7	99.7	99.7	99.7	99.7	99.3	99.6	99.6	99.8	99.7	99.7	99.8	99.6	99.8	99.7
[KJ909531] CY1111 China/chicken/2011	97.5	97.5	99.8	99.9	99.9	99.8	99.9	99.9	99.9	99.4	99.8	99.8	99.9	99.9	99.8	99.9	99.8	99.9	99.8
[KY581581] HB2015021 China/chicken/2015	97.3	97.3	99.6	99.7	99.7	99.6	99.7	99.7	99.7	99.2	99.5	99.5	99.7	99.7	99.6	99.7	99.5	99.7	99.6
[MW046297] GX18NNR1 China/chicken/2019	97.4	97.5	99.8	99.8	99.8	99.8	99.8	99.8	99.8	99.4	99.7	99.7	99.9	99.8	99.8	99.9	99.7	99.9	99.8
[JQ804915] 1105 China/muscovy duck/2011	97.5	97.5	99.8	99.9	99.9	99.8	99.9	99.9	99.9	99.4	99.8	99.8	99.9	99.9	99.8	99.9	99.8	99.9	99.8
[MW046294] GD20R1 China/chicken/2020	97.4	97.4	99.7	99.8	99.8	99.7	99.8	99.8	99.8	99.3	99.7	99.7	99.8	99.8	99.7	99.8	99.7	99.8	99.7
[AF246698] IL-74/chicken/1972	97.6	97.7	99.9	99.9	99.9	99.9	99.9	99.9	99.9	99.4	99.8	99.8	99.9	99.9	99.8	99.9	99.8	99.9	99.8
**[OK631657] USP-586 Brazil/chicken/2014**	97.5	97.6	99.9	99.9	99.9	99.9	99.8	99.9	99.9	99.4	99.7	99.7	99.9	99.8	99.8	99.9	99.7	99.9	99.8
[MF185397] SDAUR-S1 China/chicken/2017	94.4	94.4	96	96.1	96.1	96	96	96.1	96.1	95.7	95.9	95.9	96	96	95.9	96	95.9	96	95.9
[DQ003591] SNV USA/duck/1959	94.4	94.5	96.1	96.1	96.1	96.1	96	96.1	96.1	95.7	95.9	95.9	96.1	96	96	96.1	95.9	96.1	96
[KF709431] GD1210 China/chicken/2012	94.2	94.3	95.9	96	96	95.9	95.9	96	96	95.6	95.7	95.7	95.9	95.9	95.8	95.9	95.7	95.9	95.8
[KF313137] ATCC-VR775 USA/duck/1972	93.1	93.2	94.8	94.8	94.8	94.8	94.7	94.8	94.8	94.3	94.6	94.6	94.8	94.7	94.7	94.8	94.6	94.8	94.7
[GQ375848] HLJ07I China/chicken/2007	97.3	97.3	99.6	99.7	99.7	99.6	99.7	99.7	99.7	99.2	99.5	99.5	99.7	99.7	99.6	99.7	99.5	99.7	99.6
[KX278301] IBD-C1605 China/IBDV vaccine/2016	94.2	94.2	95.9	95.9	95.9	95.9	95.8	95.9	95.9	95.5	95.7	95.7	95.9	95.8	95.7	95.9	95.7	95.9	95.7

	[FJ439119] goose/3410/06	[KJ909530] SY1209	[MW046296] GX19YLR1	[KF305089] HA1101	[FJ439120] chicken/3337/05	[MW046295] GX19NNR1	[KJ909531] CY1111	[KY581581] HB2015021	[MW046297] GX18NNR1	[JQ804915] 1105	[MW046294] GD20R1	[AF246698] IL-74	**[OK631657] USP-586**	[MF185397] SDAUR-S1	[DQ003591] SNV	[KF709431] GD1210	[KF313137] ATCC-VR775	[GQ375848] HLJ07I	[KX278301] IBD-C1605
[MG471384] BJ1503 China/chicken/2015	95.5	95.4	95.5	95.4	95.5	95.2	95.5	95	95.5	95.5	95.2	95.7	95.7	92.3	92.3	92	91.6	95	91.6
[AY842951] HA9901 China/chicken/1999	95.9	95.7	95.9	95.5	95.9	95.5	95.9	95.4	95.9	95.9	95.5	96.1	96.1	92.5	92.5	92.1	91.8	95.4	91.8
[MH709124] FWPV-MN00.2 USA/chicken/2000	99.7	99.5	99.7	99.3	99.7	99.3	99.7	99.1	99.7	99.7	99.3	99.8	99.8	95.1	95.1	94.7	94.5	99.1	94.4
[KY498002] Ind/Guj-2011 India/poultry/2011	99.8	99.7	99.8	99.5	99.8	99.5	99.8	99.3	99.8	99.8	99.5	99.9	99.9	95.2	95.2	94.9	94.7	99.3	94.5
[KJ756349] 104865 USA/turkey/2014	99.8	99.7	99.8	99.5	99.8	99.5	99.8	99.3	99.8	99.8	99.5	99.9	99.9	95.2	95.2	94.9	94.7	99.3	94.5
[DQ387450] APC-566 USA/prairie chicken/2005	99.7	99.5	99.7	99.3	99.7	99.3	99.7	99.1	99.7	99.7	99.3	99.8	99.8	95.1	95.1	94.7	94.5	99.1	94.4
**[OK631658] USP-976 Brazil/chicken/2018**	99.8	99.7	99.8	99.5	99.8	99.5	99.8	99.3	99.8	99.8	99.5	99.7	99.7	94.9	94.9	94.5	94.4	99.3	94.2
[MW142017] FWPV-S Australia/vaccine/1997	99.8	99.7	99.8	99.5	99.8	99.5	99.8	99.3	99.8	99.8	99.5	99.9	99.9	95.2	95.2	94.9	94.7	99.3	94.5
[MH734528] FWPV-SD15-670.2 USA/Merriams wild turkey/2015	99.8	99.7	99.8	99.5	99.8	99.5	99.8	99.3	99.8	99.8	99.5	99.9	99.9	95.2	95.2	94.9	94.7	99.3	94.5
[MZ833509] TB01 China/Tibetan chicken/2019	99	98.8	99	98.6	99	98.6	99	98.5	99	99	98.6	98.8	98.8	94.5	94.5	94.2	93.7	98.5	93.9
[KU204702] GDBL1401 China/adult pigeon/2014	99.5	99.3	99.5	99.1	99.5	99.1	99.5	99	99.5	99.5	99.1	99.3	99.3	94.5	94.5	94.2	94	99	93.9
[KU204703] GDBL1402 China/young pigeon/2014	99.5	99.3	99.5	99.1	99.5	99.1	99.5	99	99.5	99.5	99.1	99.3	99.3	94.5	94.5	94.2	94	99	93.9
[KU641115] LN1201 China/chicken/2012	99.9	99.8	99.9	99.7	99.9	99.7	99.9	99.5	99.9	99.9	99.7	99.8	99.8	95.1	95.1	94.7	94.5	99.5	94.4
[JX912710] MD-2 China/HVT vaccine/2007	99.8	99.7	99.8	99.5	99.8	99.5	99.8	99.3	99.8	99.8	99.5	99.7	99.7	94.9	94.9	94.5	94.4	99.3	94.2
[GQ415646] HLJR0901 China/chicken/2009	99.9	99.8	99.9	99.7	99.9	99.7	99.9	99.5	99.9	99.9	99.7	99.8	99.8	95.1	95.1	94.7	94.5	99.5	94.4
[MT138121] gps222ret1 China/Cabots tragopan/2018	99.9	99.8	99.9	99.7	99.9	99.7	99.9	99.5	99.9	99.9	99.7	99.8	99.8	95.1	95.1	94.7	94.5	99.5	94.4
[MN812764] CH-GD2019 China/duck/2019	99.5	99.3	99.5	99.1	99.5	99.1	99.5	99	99.5	99.5	99.1	99.3	99.3	94.5	94.5	94.2	94	99	93.9
[MF631845] REV-TH/CBI/2013/CU-1 Thailand/chicken/2013	99.9	99.8	99.9	99.7	99.9	99.7	99.9	99.5	99.9	99.9	99.7	99.8	99.8	95.1	95.1	94.7	94.5	99.5	94.4
[MT138120] cat223ret1 China/Cabots tragopan/2018	99.7	99.5	99.7	99.3	99.7	99.3	99.7	99.1	99.7	99.7	99.3	99.5	99.5	94.7	94.7	94.4	94.2	99.1	94
[FJ439119] goose/3410/06 Taiwan/goose/2006		99.8	99.9	99.7	99.9	99.7	99.9	99.5	99.9	99.9	99.7	99.8	99.8	95.1	95.1	94.7	94.5	99.5	94.4
[KJ909530] SY1209 China/chicken/2012	99.8		99.8	99.5	99.8	99.5	99.8	99.3	99.8	99.8	99.5	99.7	99.7	94.9	94.9	94.5	94.4	99.3	94.2
[MW046296] GX19YLR1 China/chicken/2019	99.8	99.8		99.7	99.9	99.7	99.9	99.5	99.9	99.9	99.7	99.8	99.8	95.1	95.1	94.7	94.5	99.5	94.4
[KF305089] HA1101 China/chicken/2011	99.8	99.8	99.8		99.7	99.3	99.7	99.1	99.7	99.7	99.3	99.5	99.5	95.1	95.1	94.7	94.4	99.1	94.4
[FJ439120] chicken/3337/05 Taiwan/chicken/2005	99.9	99.8	99.8	99.8		99.7	99.9	99.5	99.9	99.9	99.7	99.8	99.8	95.1	95.1	94.7	94.5	99.5	94.4
[MW046295] GX19NNR1 China/chicken/2019	99.7	99.7	99.8	99.7	99.7		99.7	99.1	99.7	99.7	99.3	99.5	99.5	94.7	94.7	94.4	94.2	99.1	94
[KJ909531] CY1111 China/chicken/2011	99.9	99.8	99.9	99.8	99.9	99.8		99.5	99.9	99.9	99.7	99.8	99.8	95.1	95.1	94.7	94.5	99.5	94.4
[KY581581] HB2015021 China/chicken/2015	99.7	99.6	99.7	99.6	99.7	99.6	99.8		99.5	99.5	99.1	99.3	99.3	94.5	94.5	94.2	94	99	93.9
[MW046297] GX18NNR1 China/chicken/2019	99.8	99.8	99.8	99.8	99.8	99.7	99.8	99.6		99.9	99.7	99.8	99.8	95.1	95.1	94.7	94.5	99.5	94.4
[JQ804915] 1105 China/muscovy duck/2011	99.9	99.8	99.9	99.8	99.9	99.8	99.9	99.8	99.8		99.7	99.8	99.8	95.1	95.1	94.7	94.5	99.5	94.4
[MW046294] GD20R1 China/chicken/2020	99.8	99.7	99.8	99.7	99.8	99.7	99.9	99.7	99.7	99.9		99.5	99.5	94.7	94.7	94.4	94.2	99.1	94
[AF246698] IL-74/chicken/1972	99.9	99.8	99.8	99.8	99.9	99.7	99.9	99.7	99.8	99.9	99.8		99.9	95.2	95.2	94.9	94.7	99.3	94.5
**[OK631657] USP-586 Brazil/chicken/2014**	99.8	99.8	99.8	99.8	99.8	99.7	99.8	99.6	99.8	99.8	99.7	99.9		95.2	95.2	94.9	94.7	99.3	94.5
[MF185397] SDAUR-S1 China/chicken/2017	96	95.9	95.9	96	96	95.8	96	95.7	95.9	96	95.9	96.1	96		99.9	99.7	97.8	94.5	99.3
[DQ003591] SNV USA/duck/1959	96	96	96	96.1	96	95.9	96	95.8	96	96	95.9	96.1	96.1	99.9		99.7	97.8	94.5	99.3
[KF709431] GD1210 China/chicken/2012	95.9	95.8	95.8	95.9	95.9	95.7	95.9	95.6	95.8	95.9	95.7	96	95.9	99.8	99.8		97.4	94.2	99
[KF313137] ATCC-VR775 USA/duck/1972	94.7	94.7	94.7	94.7	94.7	94.5	94.7	94.5	94.7	94.7	94.6	94.8	94.8	97.6	97.6	97.4		94	97.1
[GQ375848] HLJ07I China/chicken/2007	99.7	99.6	99.6	99.6	99.7	99.5	99.7	99.4	99.6	99.7	99.5	99.7	99.6	95.7	95.8	95.6	94.5		93.9
[KX278301] IBD-C1605 China/IBDV vaccine/2016	95.8	95.7	95.7	95.9	95.8	95.6	95.8	95.6	95.7	95.8	95.7	95.9	95.9	99.7	99.8	99.6	97.4	95.6	

[1] Nucleotides comparison are below the diagonal line.

[2] Deduced amino acids comparison are above the diagonal line.

viruses

MDPI

Table S8. Polymorphisms in *env* gene motifs of the compared strains of REV.

MOTIF	CONSENSUS SEQUENCE	LOGO	POLYMORPHISM (ACCESSION N°)
Epitope	^{213}SVQYHPL^{219}	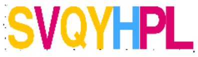	Conserved
	^{139}KTYFTAILQKTKLG^{152}	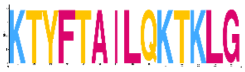	Conserved
	^{139}KTYFTAILQKTKLGS^{153}	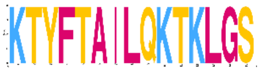	Conserved
	^{139}KTYFTAILQKTKLGSY^{154}	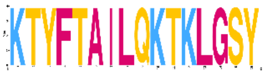	Conserved
	^{137}LGKTYFTAILQKTKLG^{152}	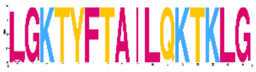	**L137M** (MG471384, AY842951)
	^{137}LGKTYFTAILQKTKLGSY^{154}	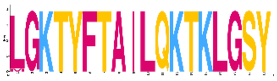	**L137M** (MG471384, AY842951)
Fusion peptide	^{402}FIPLLVGLGITGATLAGGTGL^{422}	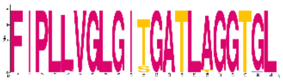	**L406P** (MG471384, AY842951), **T412S** (MF185397, DQ003591, KF709431, KF313137, KX278301, **A417T** (MG471384, AY842951), **G421E** (KY581581)
Immunosuppression domain	^{462}LQNRRGLDLLTAEQGGI^{478}	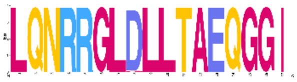	**D469G** (MW046295)
Disulfide bond in SU	^{255}CWLC^{258}	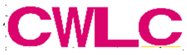	**C258S** (MZ833509)
Disulfide bond in TM	^{479}CLALQEKCC^{487}	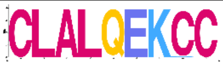	**C486R** (MW046295)

viruses

MDPI

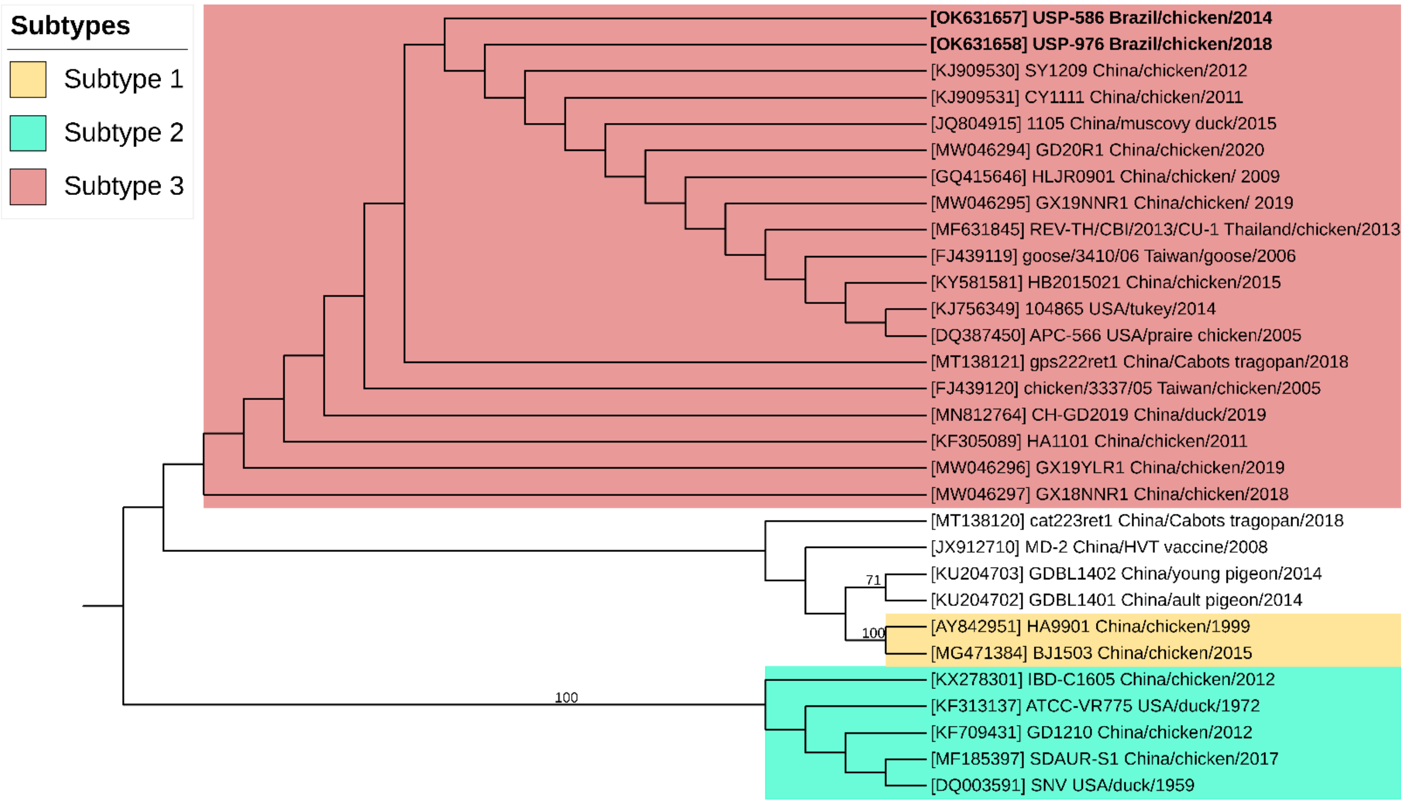

Figure S1. Phylogenetic analysis based on REV LTR region. The evolutionary tree inferred with the Maximum Likelihood method with 1000 bootstraps replications. The evolutionary distances were computed using the K80 model.

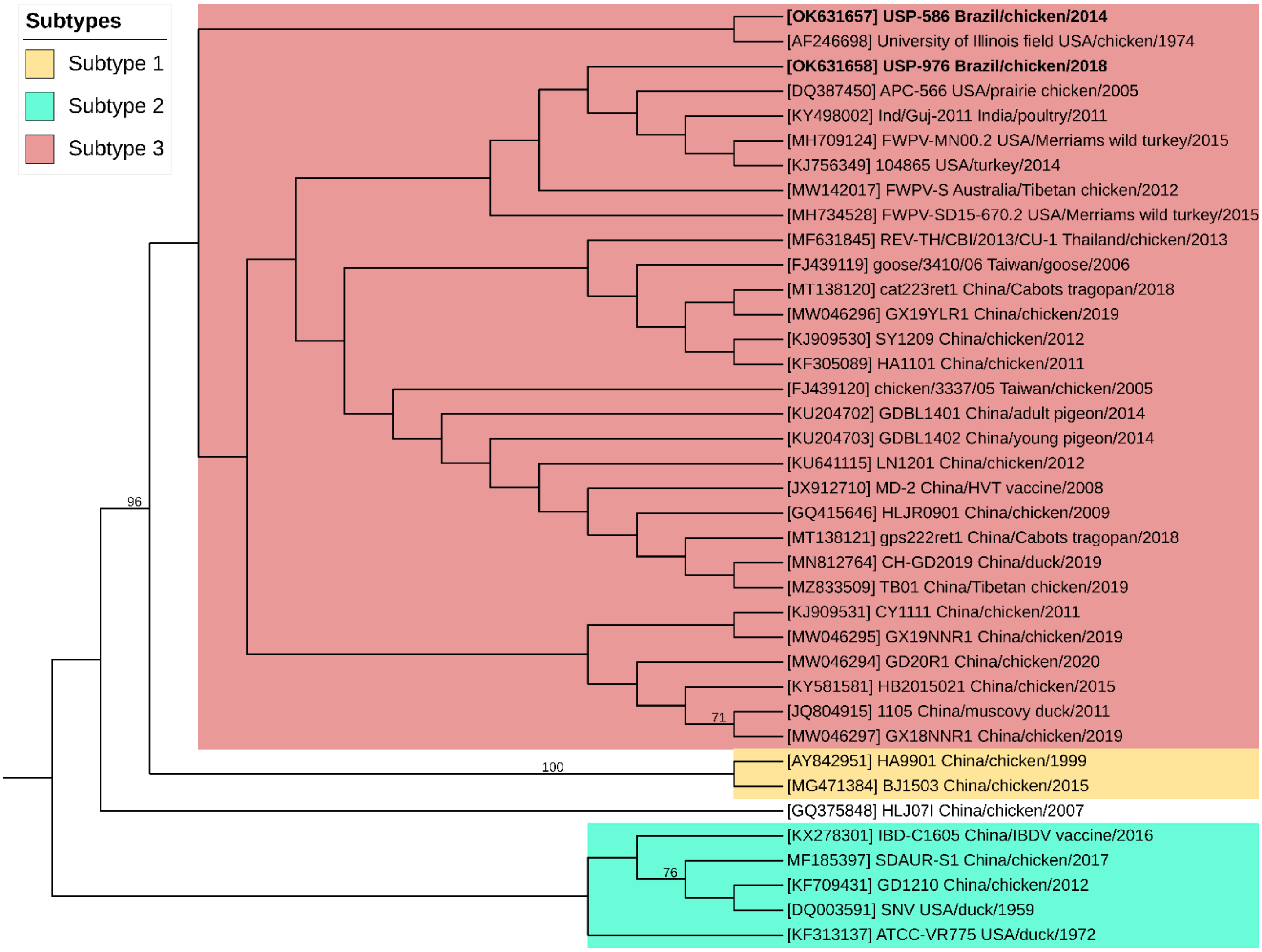

Figure S2. Phylogenetic analysis based on REV *gag* gene. The evolutionary tree inferred with the Maximum Likelihood method with 1000 bootstraps replications. The evolutionary distances were computed using the HKY + I model.

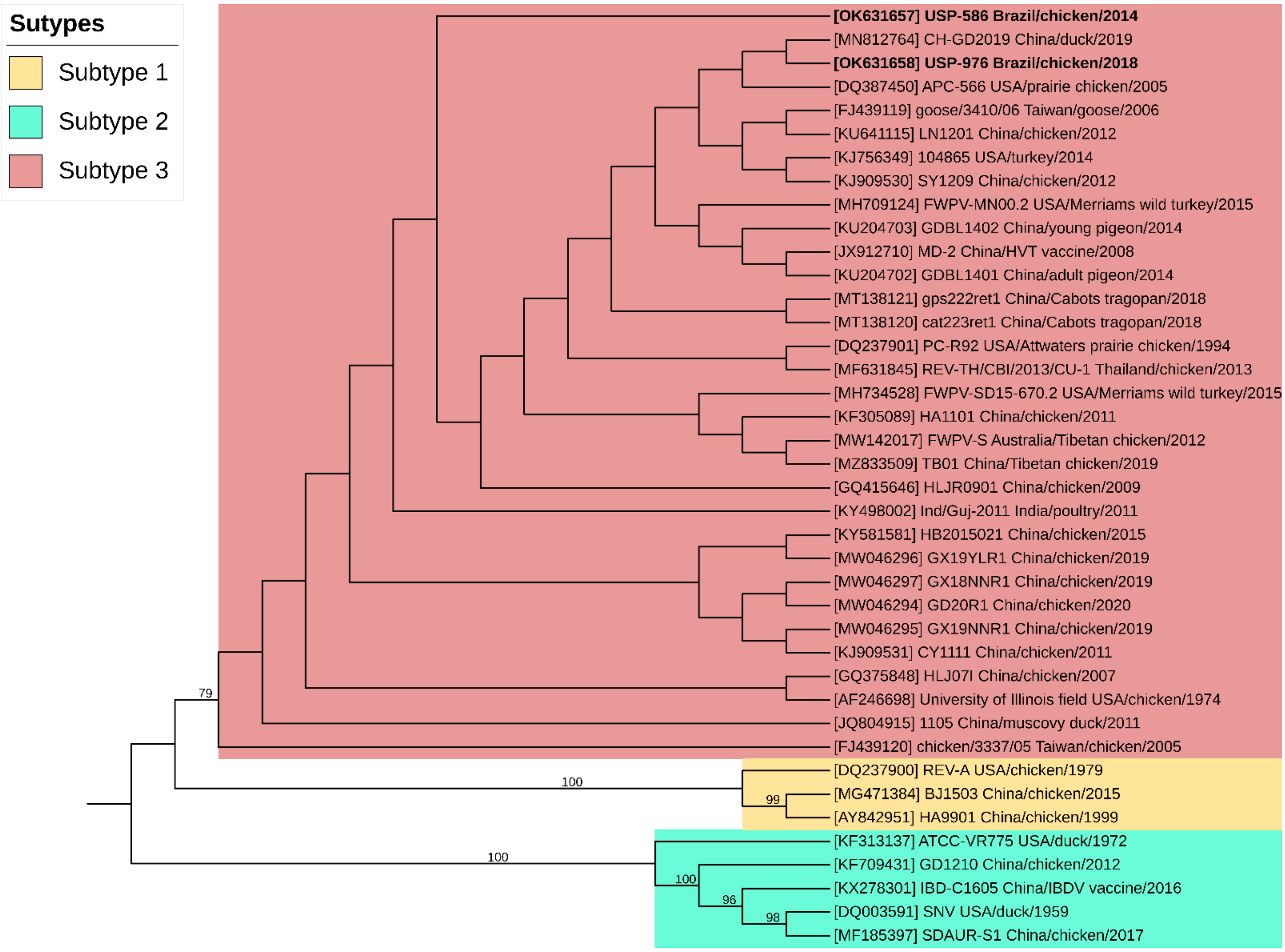

Figure S3. Phylogenetic analysis based on REV *pol* gene. The evolutionary tree inferred with the Maximum Likelihood method with 1000 bootstraps replications. The evolutionary distances were computed using the HKY + I model.

viruses

MDPI

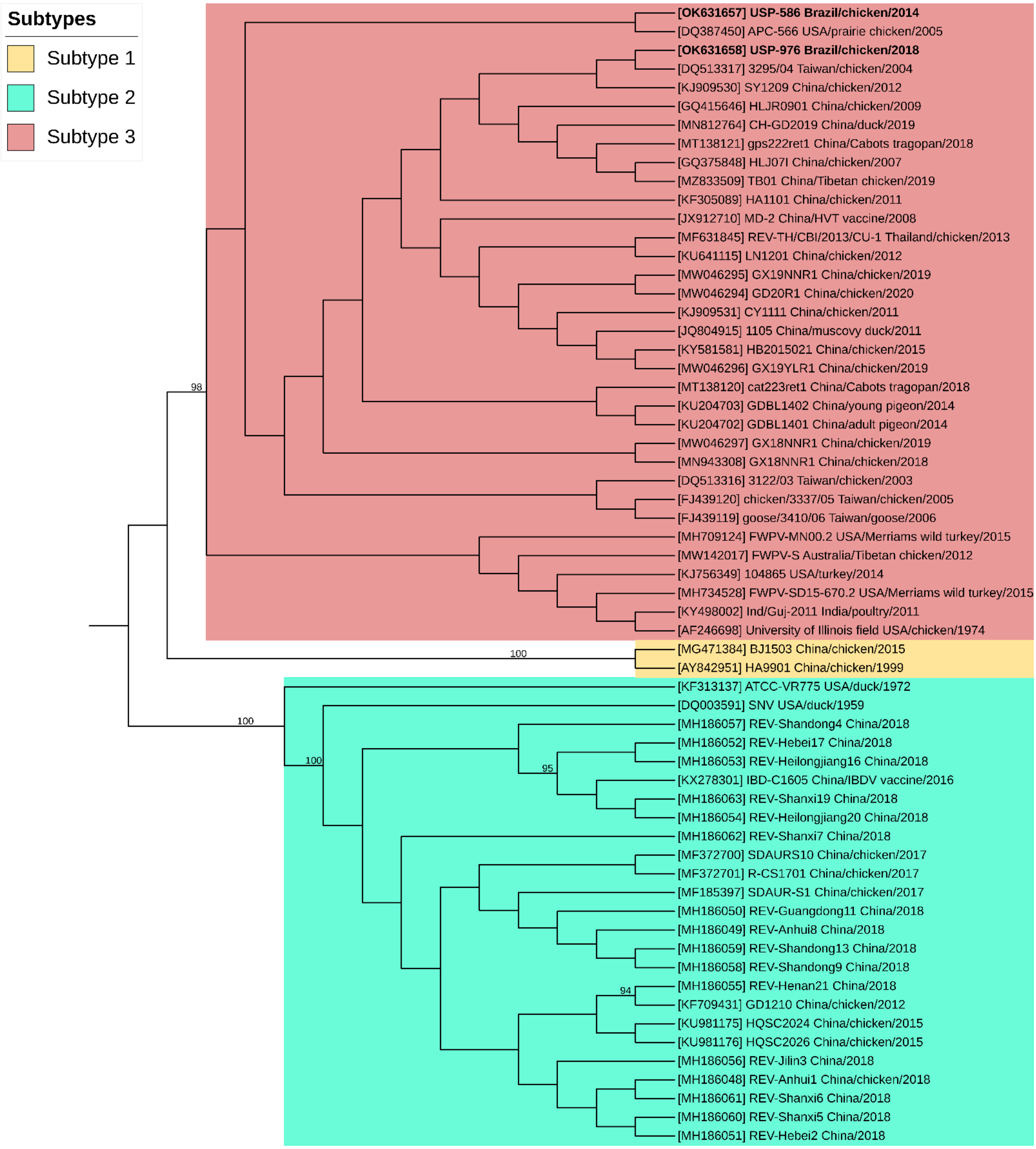

Figure S4. Phylogenetic analysis based on REV *env* gene. The evolutionary tree inferred with the Maximum Likelihood method with 1000 bootstraps replications. The evolutionary distances were computed using the HKY model.
